# The S-layer protein of a *Clostridium difficile* SLCT-11 strain displays a complex glycan required for normal cell growth and morphology

**DOI:** 10.1074/jbc.RA118.004530

**Published:** 2018-10-01

**Authors:** Emma Richards, Laura Bouché, Maria Panico, Ana Arbeloa, Evgeny Vinogradov, Howard Morris, Brendan Wren, Susan M. Logan, Anne Dell, Neil F. Fairweather

**Affiliations:** From the ‡ Department of Life Sciences, Imperial College London, SW7 2AZ London, United Kingdom,; the § Vaccine Program, Human Health Therapeutics Research Centre, National Research Council, Ottawa, Ontario K1A 0R6, Canada,; ¶ Biopharmaspec, Suite 3.1, Lido Medical Centre, St. Saviours Road, JE2 7LA Jersey, United Kingdom, and; the ‖ London School of Hygiene and Tropical Medicine, WC1E 7HT, London, United Kingdom

**Keywords:** mass spectrometry (MS), nuclear magnetic resonance (NMR), microbiology, glycoprotein structure, genetics, Clostridium difficile, surface layer, virulence, glycosylation, glycomics, glycoproteomics, cell wall

## Abstract

*Clostridium difficile* is a bacterial pathogen that causes major health challenges worldwide. It has a well-characterized surface (S)-layer, a para-crystalline proteinaceous layer surrounding the cell wall. In many bacterial and archaeal species, the S-layer is glycosylated, but no such modifications have been demonstrated in *C. difficile.* Here, we show that a *C. difficile* strain of S-layer cassette type 11, Ox247, has a complex glycan attached via an *O*-linkage to Thr-38 of the S-layer low-molecular-weight subunit. Using MS and NMR, we fully characterized this glycan. We present evidence that it is composed of three domains: (i) a core peptide–linked tetrasaccharide with the sequence -4-α-Rha-3-α-Rha-3-α-Rha-3-β-Gal-peptide; (ii) a repeating pentasaccharide with the sequence -4-β-Rha-4-α-Glc-3-β-Rha-4-(α-Rib-3-)β-Rha-; and (iii) a nonreducing end–terminal 2,3 cyclophosphoryl-rhamnose attached to a ribose-branched sub-terminal rhamnose residue. The Ox247 genome contains a 24-kb locus containing genes for synthesis and protein attachment of this glycan. Mutations in genes within this locus altered or completely abrogated formation of this glycan, and their phenotypes suggested that this S-layer modification may affect sporulation, cell length, and biofilm formation of *C. difficile*. In summary, our findings indicate that the S-layer protein of SLCT-11 strains displays a complex glycan and suggest that this glycan is required for *C. difficile* sporulation and control of cell shape, a discovery with implications for the development of antimicrobials targeting the S-layer.

## Introduction

The Gram-positive anaerobe *Clostridium difficile* remains a highly problematic bacterial pathogen, both in hospital environments and in the community (1, 2). It can cause severe gastrointestinal disease, mediated largely through the production of two toxins, TcdA and TcdB, that glycosylate host gastrointestinal GTPases resulting in damage to gut tissues and unregulated inflammatory reactions (3). Transmission of *C. difficile* infection (CDI)^8^ is through robust spores produced in the intestine as part of the normal life cycle of the bacterium (4). Vegetative *C. difficile* cells are resistant to most common antibiotics, with only a few selected antimicrobials used to treat CDI (1). Animal and clinical studies have demonstrated that broad spectrum antibiotics act as predisposing agents for CDI, through selective killing of members of the normal gut microbiota, allowing germination of *C. difficile* spores and proliferation of the vegetative cells (5–7).

**Figure 1.**
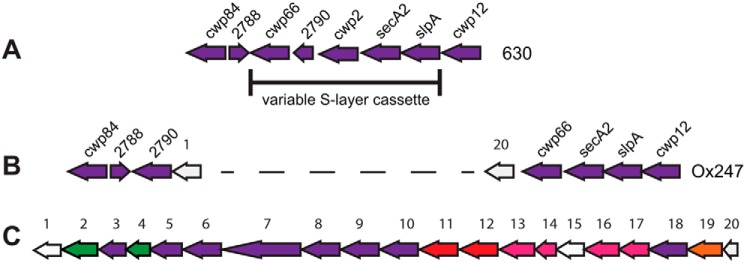
***C. difficile* S-layer and glycosylation genetic loci.** The S-layer loci of strains 630 (*A*) and Ox247 (*B*) show the relative locations of *slpA* and surrounding genes. Insertion of a 24-kb locus harboring a putative glycosylation machinery is shown in Ox247 and in more detail in *C*. The ORFs of the glycosylation locus in *C* are numbered according to Ref. 11 and are color-coded to indicate putative function: *green,* glycosyltransferases; *purple,* glycosyltransferases; *red,* ABC transporter; *pink,* rhamnose biosynthesis; *orange,* oligosaccharide ligase; *white,* no function predicted.

The cell surface of *C. difficile* has been studied in some detail. Covering the entire surface of vegetative cells is an S-layer, a proteinaceous para-crystalline array composed of S-layer proteins (SLPs) and related cell wall proteins (8, 9). The major S-layer proteins, LMW and HMW SLPs, are derived from post-translational cleavage of an ∼95-kDa precursor, SlpA (10). The *slpA* gene is encoded within a genetic locus, termed the S-layer cassette (SLC), that encodes *slpA* and the adjacent genes *cwp66*, *cd2790*, and *secA2* (Fig. 1) (11). Genetic variation in *slpA* is observed and is reflected in amino acid variation in SlpA, particularly in the LMW SLP, which shows higher sequence diversity than the HMW SLP (10).

Most archaeal species and many bacterial species present an S-layer on their outer surface (8, 12). S-layers are composed primarily of a single species of (glyco)protein covering the entire surface of the cell and present as a two-dimensional array (12, 13). No single function has been described for S-layers; rather a diversity of sequences is found that appear to accommodate a wide range of functions ranging from a selectivity barrier through various roles in pathogenesis (8, 14). In *C. difficile*, the S-layer protein SlpA is recognized by TLR4 on immune cells and may be a primary factor for the immune system to recognize and respond to *C. difficile* infection (15). In *Bacillus anthracis*, the functions of the primary S-layer proteins are unknown, but minor S-layer proteins can function in adhesion to host cells or uptake of iron and have been shown to be essential for full virulence of the bacterium (16, 17). Structurally, S-layer proteins are found to be diverse, although the three-dimensional crystal structures of only a few have been determined (18–24). Many S-layer proteins are composed of two domains: one domain anchored to the underlying cell wall and forming the two-dimensional array, and the second domain being surface-exposed and thus suitable for mediating function. In *B. anthracis* and some other species, a pyruvylated secondary cell wall polysaccharide serves as the anchor for the S-layer proteins Sap and EA1 (25), but in *C. difficile* the S-layer is noncovalently anchored to the secondary cell wall polysaccharide PSII (26).

Some S-layers carry large, surface-exposed glycans (27–29). Glycans are normally *O-*linked to the S-layer protein in bacterial species but can be *O-* or *N*-linked in archaea. The sugars found on these glycosylated S-layer proteins can be unusual and not found in eukaryotes (29). Where studied, species expressing glycosylated S-layers contain a gene locus specifying the synthesis of the glycan chain, its export through the membrane(s), and ligation to the S-layer protein (27, 29). Glycosylation has been observed at one or several residues on S-layer proteins from several species, *e.g. Geobacillus stearothermophilus* and *Paenibacillus alvei* (27) In these cases, the glycan and the S-layer protein are not co-translocated but are exported independently across the cell wall through separate mechanisms, and it is likely this is true for many other species with glycosylated S-layers (29).

In a genomics study of over 1000 *C. difficile* strains, variation in *C. difficile* SLCs was observed, and 13 SLC types (SLCTs) were identified, each encoding a distinct SlpA protein (11). One cassette type (SLCT-11) was found to contain a large 24-kb insertion that encoded a putative glycosylation locus. In SLCT-11 strains, this locus is located downstream of *secA2*, with its insertion causing a rearrangement of *cwp66* and *cd2790* and loss of *cwp2* (Fig. 1). The genes contained within this locus resemble most closely those present in other Gram-positive species that produce glycosylated S-layers, for example *G. stearothermophilus* and *P. alvei* (27). In the former species, the glycosylation gene cluster is located immediately downstream of the S-layer gene, *sgsE*, but in *P. alvei* the S-layer gene is unlinked.

In this study, we analyze the S-layer structure of the *C. difficile* SLCT-11 strain Ox247 and show conclusively that the SlpA is modified by a large glycan whose composition and structure are determined. The roles of key genes are defined by deletion analysis, and the phenotypes associated with glycosylation are investigated.

## Results

### Genetic analysis of the Ox247 glycosylation locus

The majority of *C. difficile* strains lack a genetic locus that could encode the machinery for glycosylation of surface proteins. An exception is SLCT-11 strains that contain a 24-kb gene cluster indicative of glycosylation of surface proteins (11). The putative glycosylation locus of SLCT-11 strain Ox247 is illustrated in Fig. 1. Bioinformatics analysis (Table S1) predicted 20 genes, including those encoding an initiating glycosyltransferase (*orf2*), several glycosyltransferases (*orfs 3, 6–10,* and *18*), an ABC transporter that could potentially transport a glycan to the exterior surface of the cell (*orfs 11* and *12*), a dTDP-l-rhamnose biosynthetic pathway (*orfs 13, 14, 16,* and *17*), and a protein ligase (*orf19*). Many of these genes have homology to biosynthetic pathways that synthesize, transport, and ligate glycans to S-layer proteins in Gram-positive or Gram-negative species (11). The 20 ORFs within the glycosylation locus are transcribed as a polycistronic operon with the adjacent genes *CD2790* (downstream) and *cwp66* (upstream) (data not shown).

**Figure 2.**
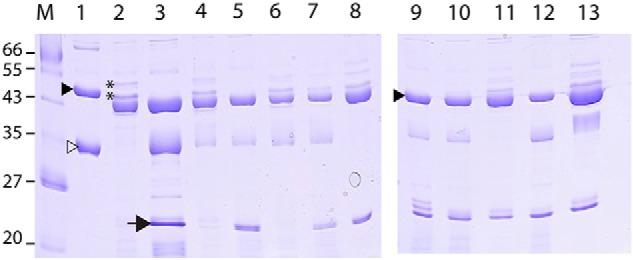
**S-layer proteins of *C. difficile* Ox247 and mutants.** Strains were cultured in BHIS broth, and the S-layer was prepared using low pH glycine extraction. The LMW (▷) and HMW (▶) S-layer proteins of strain 630 are indicated. The 20-kDa LMW SLP (→) predicted in Ox247 is not found in the WT strain, but is found in some but not all mutants. The ∼45-kDa bands in Ox247 analyzed by MS are indicated (**). *M*, *M*_r_ markers; *lane 1*, 630; *lane 2*, Ox247 WT; *lane 3*, *orf2::erm*; *lane 4*, *orf2::erm* (pAAM008); *lane 5*, *orf3::erm*; *lane 6*, *orf2::erm* (pEJR004); *lane 7*, *orf4::erm*; *lane 8*, *orf4::erm* (pEJR007); *lane 9*, *orf7::erm*; *lane 10*, *orf16::erm lane 11, orf16::erm* (pEFR009); *lane 12*, *orf19::erm*; and *lane 13*, *orf19::erm* (pAAM011).

To investigate whether this locus was involved in modification of the S-layer, mutants were constructed in Ox247 using the ClosTron mutagenesis system that inactivates genes through targeted insertion of an antibiotic resistance cassette (30). The *orf2, orf3, orf4, orf7, orf16,* and *orf19* genes were mutated in this way using an erythromycin resistance marker. S-layer proteins from Ox247 and the mutants were prepared and analyzed by SDS-PAGE (Fig. 2).

S-layer proteins from WT Ox247 can be compared with those from 630, a well characterized *C. difficile* strain. In contrast to the two predominant SLPs seen in 630, migrating at ∼43 kDa (HMW SLP) and ∼35 kDa (LMW SLP), only one highly stained band is seen in Ox247, migrating at ∼42 kDa (Fig. 2). A number of minor bands were also evident both above and below the 42-kDa band in Ox247. Sequence analysis of Ox247 SlpA predicts two SLPs formed from the cleavage of the mature SlpA protein, a HMW SLP of ∼45 kDa and a LMW SLP of ∼20 kDa. The size of the LMW SLP is considerably smaller than those observed and predicted in other *C. difficile* strains. However, this ∼20-kDa species is clearly absent from the WT Ox247 S-layer extract.

In contrast, all mutants (*orf2*, *orf3*, *orf4*, *orf7*, *orf16,* and *orf19*) produced a band at ∼20 kDa, which was shown by MS to be the LMW SLP (see below). This strongly suggests that the genetic locus does indeed encode a glycosylation pathway that modifies SlpA and that disruption of the pathway results in the appearance of the predicted ∼20-kDa LMW SLP. Comparison of the banding pattern of Ox247, the *orf2* mutant, and the mutant complemented by a plasmid expressing Orf2 reveals a lack of several high-molecular-weight bands above the HMW SLP (∼44 kDa) in the mutant. These bands were predicted to contain glycosylated forms of the ∼20-kDa LMW SLP. Complementation of the mutants was either complete, restoring the WT banding pattern (*orf2* and *orf3*), or incomplete (*orf16* and *orf19*), or was not possible (*orf7*) due to the inability to construct a stable plasmid in *Escherichia coli*.

### Discovery of complex S-layer glycosylation: Glycoproteomic and glycomic MS analyses

**Figure 3.**
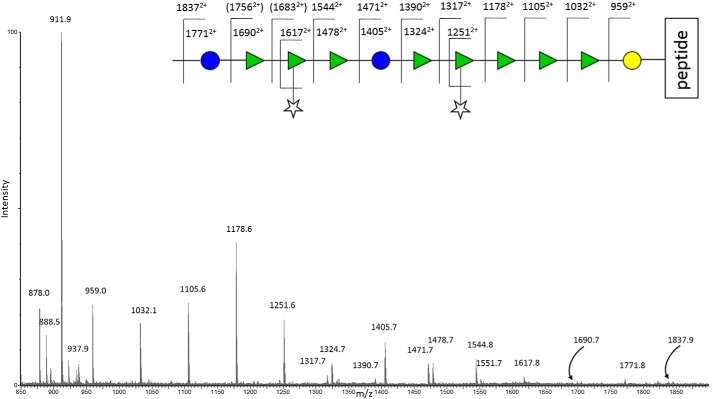
**Pattern of doubly-charged ions observed in the tryptic digest Q-TOF LC-ES-MS data set showing in-source fragmentation of a glycopeptide, the peptide component of which is observed at 878.0^2+^.** Doubly charged ions corresponding to sugar mass intervals linked to this peptide are shown in the *cartoon* above the spectrum. These data show evidence for a minimum oligosaccharide substitution of 11 residues and a sequence (starting from the reducing (peptide-attached) end) of Hex-dHex-dHex-dHex-dHex(Pent)-dHex-Hex-dHex-dHex(Pent). See the text for details of the assignments.

Unequivocal evidence for glycosylation of the S-layer was determined by glycoproteomic mass spectrometric strategies applied to the analyses of the S-layer proteins (31–34). S-layer extracts from WT and mutants were purified by gel electrophoresis, in-gel digested with trypsin, and analyzed by nano-LC coupled to electrospray MS (35–38). MS data on signals eluting between 37 and 40 min for the band at 42 kDa from WT Ox247 (Fig. 3) showed an interesting pattern of doubly charged ions, starting at *m*/*z* 878 (the signal at *m*/*z* 911.9 derives from a different peptide), separated from other prominent signals by intervals corresponding to sugars (*m*/*z* 81 for hexose (Hex), *m*/*z* 73 for deoxyhexose (dHex), and *m*/*z* 66 for pentose (Pent)). The series can be interpreted as beginning with the tryptic peptide at *m*/*z* 878 and extending by a hexose to *m*/*z* 959, by cleavage at the glycosidic bond with hydrogen rearrangement and charge retention on the reducing end fragment, followed by four dHex intervals (to *m/z* 1032, 1105, 1178, and 1251). Following the *m*/*z* 1251 signal, the pattern then deviates with two possible cleavage fragments, which indicates branching. The next increment is Pent to *m*/*z* 1317 or dHex to 1324. A common phenomenon in the MS/MS of branched saccharides is the β-elimination of side chains, sometimes giving the strongest signals not at the branching point itself, but at later cleavages. The sugar series then continues from *m*/*z* 1324 with a further hexose residue to *m*/*z* 1405, but this signal is also a pentose difference from *m*/*z* 1471. This spectrum therefore affords two possible interpretations, either with a pentose branch at the *m*/*z* 1251 (4th dHex) cleavage position to give an overall *m*/*z* of 1317, or at the Hex *m*/*z* 1471 position to give *m*/*z* 1405 by β-elimination of the pentose. The *m*/*z* 1317 is a crucial signal here, in that it cannot easily be rationalized mechanistically as deriving from the latter (Hex–Pent) possible branching series, although it can be interpreted as locating the branching point on the fourth dHex from the reducing end of the structure, with Pent loss to 1251 (see Fig. 3). *m*/*z* 1324 is then seen to derive from pentose loss from the signal at 1390 in Fig. 3. This branching substitution is suggested and proven by the NMR study (see below), and the overall assignment of the glycopeptide MS/MS spectrum in Fig. 3 shows that the oligosaccharide chain continues out to higher mass (no nonreducing end capping observed) via signals at *m*/*z* 1478, 1544, 1617, 1690 1771, and 1837 (assignments shown in Fig. 3 schematic) giving a minimal length for this novel oligosaccharide glycoprotein conjugate of 13 sugar residues at a single point of peptide *O-*linked substitution. The doubly-charged fragment ion at *m*/*z* 878 itself, together with its subfragments, established this as a peptide with the sequence DILAAQNLTTGAVILNK, which corresponds to residues 29–45 of the low-molecular-weight subunit of the S-layer protein. Other ions in the above series selected for MS/MS analysis by the data-dependent software at this time point in the LC-MS trace were the *m*/*z* 959, 1032, 1105, and 1178 glycopeptide fragment signals, and those data also confirmed the sequence conclusions shown in Fig. 3.

To investigate the biosynthesis of this unique type of glycopeptide found in the WT sample, the S-layers of the *orf2*, *orf3,* and *orf7* mutants were subjected to the same glycoproteomic strategy. All gave gel bands near 20 kDa (Fig. 2). In the case of the *orf2* mutant, this band was found to contain the unmodified LMW SLP. The bands near 20 kDa from the other mutants were found to contain glycosylated LMW SLP. The glycan chains ranged in size from two sugar units, specifically dHexHex (*orf3* mutant), up to a heptasaccharide Pentd-Hex_5_Hex seen in the *orf7* mutant (Fig. S1, *A* and *B*).

To gain a better understanding of what was clearly a larger structure than shown by the electrospray MS and MS/MS data in Fig. 3, full profiling of the S-layer glycoprotein using a more comprehensive range of MS techniques was employed (i) to define the precise site of attachment of the glycan chain to the S-layer proteins, (ii) to determine the carbohydrate composition and linkage of the glycan moieties observed in the MS/MS as Hex, dHex, and Pent, and (iii) to determine the overall size and sequence of the novel oligosaccharide component of this glycoconjugate. The S-layer glycoprotein sample was therefore prepared for study using different strategies and a range of analytical MS techniques, including GC-MS, MALDI-TOF MS, MS/MS, Q-TOF ES-MS, and MS/MS, also incorporating electron transfer dissociation (ETD) (39), as well as elimination, chemical derivatization, and GC-MS methods following hydrolysis.

#### 

##### Site of attachment

**Figure 4.**
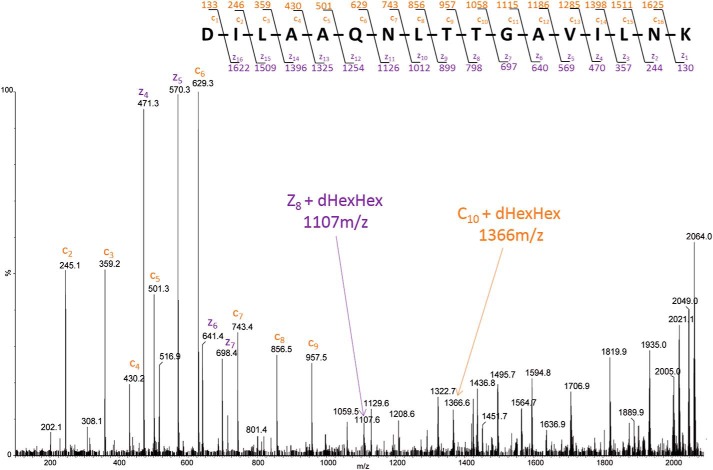
**ETD MS/MS spectrum of *m*/*z* 516.55^4+^ derived from the *C. difficile orf3::erm* mutant.** Peptide fragmentation provides strong evidence for the sequence DILAAQNLTTGAVILNK. c-ions are labeled in *orange* and z-ions in *purple*. Following the known ETD mechanism, the cleavage of the N–Cα bond occurs producing C-type (N-terminal) and Z-type (C-terminal) fragments. The mass difference between two adjacent c or z ions provides the mass and identity of the amino acid residue and any substitution, which here identifies the glycan as being attached to threonine 38 (see the text).

The ETD MS/MS spectrum of the glycopeptide [M + 4H]^4+^ (*m*/*z* 516.55) at 25 min, belonging to the 20-kDa band of the *orf3* mutant, is shown in Fig. 4. From this spectrum, there is excellent evidence for the substitution of dHexHex on Thr-38 of the sequence determined, supported by several fragment ions. First, considering the “c” series, there is no signal at *m*/*z* 1265 (c_9_ + dHexHex), but *m*/*z* 957 (free c_9_) is strong, and there is a strong peak at *m*/*z* 1366 that corresponds to c_10_ + dHexHex (1058 + 146 + 162). Second, coming from the C terminus of the peptide, the free z_8_ fragment ion is absent (*m*/*z* 798), but the glycosylated signal is seen at *m*/*z* 1107 (z_8_ + 1 + dHexHex) (Fig. 4).

##### Sugar composition and linkage

We next examined the *O-*linked glycan chain decorating the LMW SLP of *C. difficile* Ox247 via GC-MS to attempt to determine the sugar composition and, if possible, the linkages. WT Ox247 S-layer protein was extracted and purified by dialysis, reductively eliminated, and hydrolyzed into monosaccharides. Alditol acetate derivatives of the samples were then prepared and analyzed by GC-EI-MS and assigned by comparison with standards, using the GC retention times as well as the specific MS fragmentation patterns of each monosaccharide found (40). From these data, the *O-*glycan of the S-layer glycoprotein was found to contain principally rhamnose, with lesser amounts of ribose, glucose, and a small amount of galactose (Fig. S2). For a complex natural product, such as that found here for the S-layer glycan of *C. difficile* Ox247, containing a mixture of related polymeric components ranging up to approximately 50 sugar residues or more (see below), it was not thought useful to make quantitative calculations of relative ratios. An attempt was then made to carry out linkage analysis on the β-eliminated oligosaccharide, following permethylation, by the standard hydrolysis and re-acetylation method to produce partially methylated alditol acetate. Examination of the mass spectra of each peak eluting from the GC column permitted assignment by comparison with the publicly available database on the Complex Carbohydrate Research Center website (University of Georgia, Atlanta). Positive assignments were possible for terminal ribose, 3-linked rhamnose, 3,4-linked rhamnose, and 4-linked glucose (data not shown), although other unassigned small signals were also observed.

The data support the basic structural conclusions in the MS data set of Fig. 3 regarding the presence of hexose (mainly Glc but also Gal), deoxyhexose (only rhamnose), and pentose (only ribose) units in the oligosaccharide, including the facile loss of terminal pentose-branching units, but apart from the presence of some weak noninterpreted peaks, there were no further clues in the composition and linkage data as to the missing unit(s) needed to identify the nonreducing end structure.

##### Mass and sequence of the oligosaccharide

**Figure 5.**
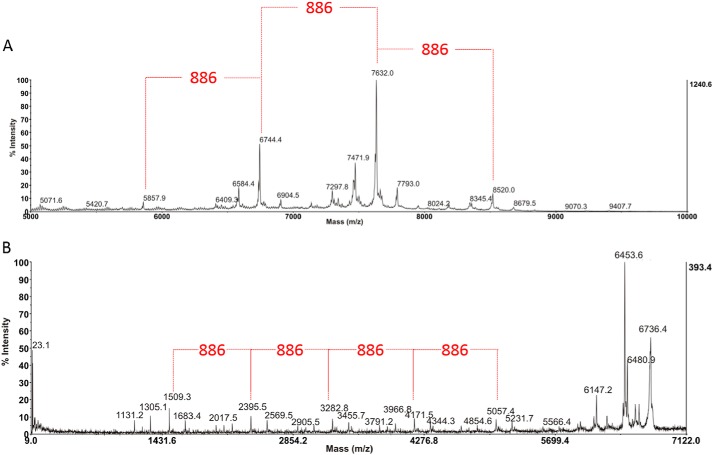
*A,* MALDI-TOF MS spectrum from *m*/*z* 5000 to 10,000 of the permethylated glycan isolated from the S-layer protein of *C. difficile* Ox247. The data provide strong evidence for the presence of a long glycan chain of some 50 sugar residues, containing a repeating pentasaccharide unit of 886 Da within its structure (highlighted in *red*), corresponding to a Glc·Rha_3_·Rib composition, and leading to different polymer lengths. For details see the text. *B,* MALDI TOF/TOF MS/MS spectrum from *m*/*z* 9 to 7122 of the signal centered around *m*/*z* 6744 in *A* which in the calibrated MS/MS spectrum is appearing with a peak-top mass of 6736. Differences of hexose, deoxyhexose, and pentose are visible throughout the spectrum, and the sequence can be built up from the reducing end fragment (corresponding to the peptide-attached oligosaccharide sequence determined from the ES-MS data in Fig. 3) as described in the text to the Rha-Glc cleavage at *m*/*z* 1509. From there, intervals of a repeating pentasaccharide sequence (886 Da) are clearly determined to *m*/*z* 2395 and higher masses toward the nonreducing end of the structure. Except for unassigned losses from the molecular ion, the reducing end moiety remained elusive at this stage of the study.

Having discovered a novel long–chain *O-*linked oligosaccharide on LMW SLP together with its precise site of attachment in the protein sequence, the objective was then to define the size of this unusual structure, including its nonreducing end and overall sequence, and for this purpose the oligosaccharide was first removed from the protein backbone by β-elimination. The sample was then derivatized by permethylation to allow examination by MALDI-TOF MS and MS/MS (41, 42). Interestingly, and surprisingly, in the MS spectrum coming from the 75% acetonitrile fraction of the WT sample acquired using the TOF/TOF instrument in linear MS mode (Fig. 5*A*), it is possible to see that the glycan chain decorating the S-layer protein of *C. difficile* Ox247 is much longer than the electrospray (ES-MS) data had indicated (in Fig. 3), probably due to the very low internal energy transfer associated with MALDI ionization preserving the higher mass structures. In fact, from the MALDI data in Fig. 5*A* there is evidence of a long glycan chain of the order of 50 sugar residues, with abundant high-mass signals around *m*/*z* 6744 and 7632, but with the highest readily visible peak at *m*/*z* 8520 (peak top), with weaker higher mass signals indicating that even that is not the limit of the structure of this molecule. Note also the weak satellite signals 160 Da lower and higher than the main ones, possibly indicating some heterogeneity with species lacking a ribose or carrying an additional one, because ribose has this mass difference when permethylated. Overall, these MS data illustrate a repeating pentasaccharide unit of mass 886 Da, corresponding to a permethylated [Hex·Rha_3_·Rib]*_n_* composition where the repeat number “*n*” varies, leading to different polymer lengths.

Several MS/MS experiments were then carried out by MALDI TOF/TOF with refined mass calibration to define the sequence, and in these spectra the presence of certain “starting numbers” and fragment ion series are clear, as seen for 6744 in Fig. 5*B*. For example, in the low mass range of the spectrum, the ion at *m*/*z* 1509 is the more abundant fragment and is separated from *m*/*z* 1305 by 204 atomic mass units (a permethylated Glc or Gal mass difference), which in turn is separated from *m*/*z* 1131 by 174 atomic mass units (a Rha difference) below that. These data, combined with knowledge of sugar fragmentation mechanisms and the fact that MALDI ionization of permethylated oligosaccharides under these conditions produces M + Na^+^ quasi-molecular ions, together also with the fact that the elimination strategy used to free the oligosaccharide from the peptide backbone leads to the production of an open-ring reducing-end residue, then allowed a test calculation to determine whether these signals came from the nonreducing or the reducing end of the overall molecular structure. In this way, *m*/*z* 1509 could be assigned to a reduced and sodiated Glc/Gal_red_–Rha_5_–Rib–Glc/Gal fragment ion, showing that (*a*) it is the reducing end fragment (attached originally to the peptide via the hexose) and (*b*) it corresponds to the first 8 residues of the 13-residue structure discovered in the Q-TOF ES-MS/MS experiment seen in Fig. 3, which was assigned as the branched structure Hex–dHex–dHex–dHex–dHex(Pent)-dHex–Hex-dHe-x-dHex(Pent)–dHex-Hex. Continuing the interpretation of the MS/MS spectrum in Fig. 5*B* from the *m*/*z* 1509 ion toward higher mass, a series of signals are found between that and the next most abundant fragment at *m*/*z* 2395, corresponding to a mass difference of 886.4 Da via *m*/*z* 1683, 2017, and 2191 assigned to a sequence of Rha-Rha(Rib)–Rha–Glc/Gal. This pentasaccharide pattern, created by a cleavage between Rha and Glc (the most abundant hexose in the compositional analysis), which would appear to be a preferred fragmentation pathway, then continues in further increments of 886 mass unit increments to *m*/*z* 3282, *m*/*z* 4171, and *m*/*z* 5057 generating a polymer chain for this particular parent ion mass (6744 in linear MS mode). Note that the mass sufficiency and predominant isotope “peak top” labeled by the instrument software, which is often several mass units higher than ^12^C, must be accounted for in the mass annotations observed in such spectra at higher masses. From these data, and the MS/MS analysis of other significantly strong signals in the MALDI MS spectrum, which themselves differ by this 886 repeating sugar units, it is clear that the novel oligosaccharide discovered here is in fact a series of closely related repeat polymer structures spanning a considerable mass range, beyond 5000 Da.

Turning to the quasi-molecular ion region of the MS/MS spectrum in Fig. 5*B*, where the “peak top” mass of the quasi-molecular ion (M + Na^+^) is corrected to 6736 Da, the losses observed do not fit for the now-established reducing-end structure and must therefore be coming from the unknown structure at the nonreducing end of the molecule. Auto-peak labeling at these very high masses depends on small fluctuations in ^13^C isotope abundances, but averaging the data from this parent ion (6736) and the MS/MS spectrum of the next higher polymer in the series shows that the losses seen are principally −255 (to 6481), −283 (to 6453), and −589 (to 6147), where the 28-atomic mass unit difference between the 6481 and 6453 could be assigned to a probable ring cleavage counterpart (containing C-1 and the ring oxygen atoms of the terminal sugar) to the glycosidic bond cleavage expected for *m*/*z* 6453. These losses were not interpretable on the basis of the standard sugars found in the composition analysis, and therefore the nonreducing end group could not be assigned from the MS data alone. To characterize the precise nature of the nonreducing end moiety, and to specify the absolute stereochemistry and linkages of the oligosaccharide, the structure was then examined by NMR spectroscopy.

### NMR analysis of S-layer glycan

To obtain sufficient SLP glycan for NMR analysis, 36 mg of SLP protein was extracted from bacterial cells using glycine buffer (43). The SLP protein extracts were extensively digested with proteinase K for 48 h, and small nonglycosylated peptide fragments were removed using Amicon filtration (3000 Da cutoff). Digested material, which was retained in the supernatant (>3000 Da), was collected and lyophilized. Further purification of this larger molecular weight, soluble material was made by applying the sample to a Hitrap Q anion-exchange column, and fractions were eluted with 0–1 m gradient of NaCl. Fractions were desalted on Sephadex 15, and glycan-containing fractions were identified by ^1^H NMR. Two glycan-containing fractions were obtained, one fraction eluted in water at the beginning of the NaCl gradient (PS-1), and a second fraction eluted later in ∼0.2 m NaCl (PS-2).

2D NMR spectra (COSY, TOCSY, ROESY, ^1^H–^13^C HSQC, ^1^H–^13^C HMBC, ^1^H–^31^P HMQC) were recorded for PS-1 and PS-2. PS-1 was identical to a previously studied *C. difficile* polysaccharide (PSII) (data not shown) (44). The second glycan was of unique structure with pentasaccharide repeating units and distinct fragments at both reducing and nonreducing ends.

**Figure 6.**
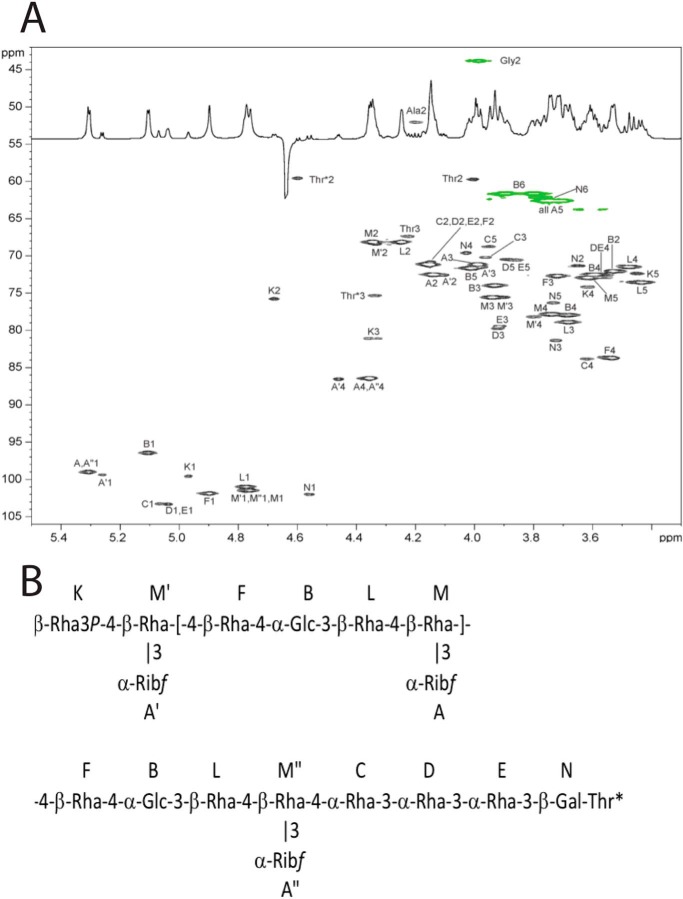
*A,* ^1^H–^13^C HSQC spectrum of the polysaccharide from S-layer protein SlpA (PS-2). CH signals are *black,* and CH_2_ signals are *green. B,* structure of PS-2.

NMR signals were completely assigned for PS-1 (data not shown) and PS-2 (Table 1 and Fig. 6*A*). Monosaccharides were identified by COSY, TOCSY, and NOE spectroscopy cross-peak patterns and ^13^C NMR chemical shifts. Connections between monosaccharides were determined from transglycosidic NOE and HMBC correlations. For PS-2, the following NOE correlations from H-1 of the monosaccharides were observed: A1, M1,2,3; A′1, M′2,3; B1, L1,2,3; B1, M6; C1, D3; D1, E3; E1, N3; K1, M′4,6; F1, B2,3,4; M1, F4,6; M′1, F4,6; M″1, C4; L1, M4,6; N1, Thr*3. HMBC correlations were in agreement with NOE data: A1, M3; A′1M′3; A″1M″3; B1, L3; C1, D3; D1, E3; E1, N3; K1, M′4; F1, B4; L1, M4; M1, F4; M′1, F4; M″1, C4; N1, Thr-3. These observations allowed the assembly of the structure presented in Fig. 6*B*.

**Table 1:** **NMR data for the S-layer polysaccharide PS-B (ppm from acetone, 600 MHz, 35 °C)**

	H/C-1	H/C-2	H/C-3	H/C-4	H/C-5	H/C-6
Ribf A	5.31	4.14	3.99	4.35	3.70; 3.76	
	99.0	72.5	71.1	86.4	62.6	
Ribf A′	5.26	4.11	3.97	4.46	3.74; 3.74	
	99.4	72.6	71.5	86.5	62.6	
Ribf A″	5.30	4.14	3.99	4.35	3.70; 3.76	
	99.0	72.5	71.1	86.4	62.6	
α-Glc B	5.10	3.59	3.93	3.68	4.01	3.80; 3.89
	96.5	72.5	73.9	77.9	71.6	61.6
α-Rha C	5.06	4.14	3.96	3.62	3.95	1.32
	103.3	71.2	70.2	83.8	68.7	17.9
α-Rha D	5.04	4.15	3.92	3.56	3.86	1.31
	103.3	71.2	79.7	72.5	70.5	17.9
α-Rha E	5.03	4.17	3.91	3.58	3.88	1.30
	103.3	71.2	79.4	72.5	70.4	17.9
β-Rha F	4.90	4.15	3.72	3.54	3.54	1.34
	101.9	71.2	72.7	83.7	72.1	17.9
β-Rha K	4.97	4.68	4.34	3.62	3.45	1.33
	99.6	75.8	81.1	74.1	72.3	17.9
β-Rha L	4.77	4.24	3.68	3.44	3.43	1.34
	101.0	68.1	78.9	73.5	73.6	17.9
β-Rha M	4.76	4.34	3.94	3.74	3.62	1.38
	101.4	68.1	75.5	77.8	72.9	18.2
β-Rha M′	4.78	4.33	3.90	3.80	3.62	1.40
	101.4	68.4	75.5	78.2	72.9	18.2
β-Rha M″	4.77	4.34	3.94	3.74	3.62	1.38
	101.4	68.1	75.5	77.8	72.9	18.2
β-Gal N	4.56	3.65	3.72	4.03	3.73	3.78; 3.78
	102.0	71.3	81.3	69.6	76.3	62.1
Thr*		4.60	4.34	1.29		
		59.5	75.3	17.0		
Thr		4.00	4.22	1.34		
		59.7	67.3	19.9		
Gly		3.98; 3.98				
		43.8				
Ala		4.20	1.33			
		52.0	18.8			

For PS-2, signals from the internal repeating units as well as reducing and nonreducing ends of the polymer were visible. The reducing end was occupied by a tetrapeptide containing Ala, Gly, and two Thr residues. The reducing end sequence of monosaccharides -3-α-Rha-3-α-Rha-3-β-Gal- was different from that of the repeating units. Terminal Gal was linked to one of the Thr residues of the peptide, as assignable from NOE and HMBC data. This is a typical *O-*linkage of glycan to protein in S-layer proteins.

**Figure 7.**
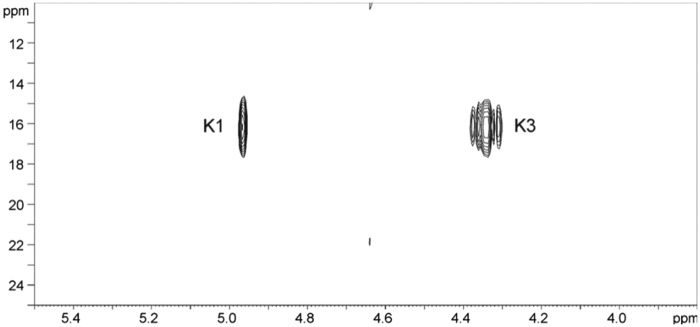
**^1^H–^31^P HSQC spectrum of PS-2.**

The nonreducing end of this unique glycan was occupied by a β-Rha residue K. This monosaccharide had ^1^H and ^13^C signals of H,C-2,3 strongly shifted to low field when compared with the expected values for nonsubstituted β-Rha (Table 1). The ^31^P spectrum contained a signal at 16.2 ppm, correlating in ^1^H–^31^P HSQC to K1 and K3 but not to K2 (Fig. 7). H-3 signal had additional 18 Hz splitting due to H–P coupling. The nature of this phosphate remained unclear at this stage; In addition the ∼10 ppm downfield shift of H-3 signal and the huge coupling constant with ^31^P are also difficult to explain. Particularly intriguing is the correlation H-1–P with no trace of H-2–P correlation. At this point, we were unable to assign a definitive structure for this phospho-derivatized rhamnose nonreducing end residue but made a tentative 3-phospho-Rha assignment (Fig. 6*B*).

### Correlation of the mass spectrometry and NMR data

The detection of a phosphate proton resonance in the NMR then allowed a re-examination of the MALDI MS/MS data to correlate masses observed with a possible phosphate-substituted rhamnose nonreducing end moiety. First, in the interpretation of the data in the quasi-molecular ion region of the permethylated sample in Fig. 5*B*, the loss of 283 mass units to give *m*/*z* 6453 can now be rationalized as the loss of a dimethyl-phospho-rhamnose terminal unit via glycosidic bond cleavage with hydrogen transfer. *m*/*z* 6481 is the ring-cleavage fragment retaining H–C=O on the reducing end referred to earlier. The remaining abundant ion in this region at *m*/*z* 6147 is then assigned as the equivalent ring fragment to 6481 at the next residue, which is assigned as a branched Rha-(Rib), 334-atomic mass unit difference.

Importantly, this assignment of a terminal Rha-phosphate moiety (which from sub-fragment data could be positioned at the 2- or 3-position in the ring) did not correlate with the overall mass data coming from both the MALDI MS of permethylated derivatives or from the data on the intact glycopeptide itself, and this now required explanation. Fig. 9 shows the negative ion MALDI MS/MS spectrum of the native glycopeptide isolated by proteinase K digestion in preparing the NMR sample above. This spectrum was determined by collisionally activated decomposition of the parent ion observed at *m*/*z* 6563.6 (peak top) for the [M − H]^−^ species. In arithmetic terms, this would correspond to a ^12^C mass of 6560.6 for the glycopeptide. Because in other (electrospray) data (not shown) we had definitively assigned the peptide portion of the molecule as the sequence TTGA (with no ragged ends), the theoretical ^12^C mass for the glycopeptide, containing a terminal Rha-phosphate and with the repeat unit *n* = 7, is calculated as 6578.4. Although mass accuracy in this difficult type of high-mass negative ion MS/MS experiment on small amounts of material can be questionable, within plus or minus a mass unit or so, it is nevertheless clear that the intact mass data on the native glycopeptide does not correlate with a phosphate substitution of the terminal rhamnose and is roughly 18 mass units lower than the theoretical mass overall.

**Figure 8.**
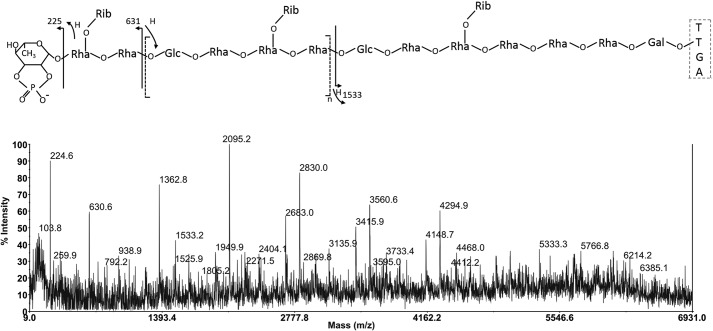
**Negative ion MALDI TOF/TOF MS/MS spectrum of the native glycopeptide of Ox247 isolated by proteinase K digestion.** The data show the detailed fragmentation produced from a quasi-molecular ion signal found in the MS spectrum at (peak-top) *m*/*z* 6563.6, and clearly identify (i) a nonreducing end structure 18 mass units below that expected for phospho-rhamnose and (ii) an intact mass some 18 atomic mass units lower than expected for the overall predicted structure in Fig. 7. These data suggest a cyclophospho-rhamnose nonreducing terminus. For detailed interpretation see the fragmentation cartoon above the spectrum and the text.

Fig. 8 explains the solution to this conundrum, via interpretation of the important major fragment ions at *m*/*z* 225 and 631 in the MS/MS spectrum of 6564 [M − H]^−^. *m*/*z* 225 actually provides the evidence for the source of the 18-atomic mass unit difference between experimental and theoretical masses discussed above, because it is 18 atomic mass units below what would be expected for a phosphate-substituted rhamnose residue. This mass was assigned as cyclophospho-rhamnose as shown in Fig. 8, and the fragment ion itself derives from glycosidic bond cleavage with retention of the glycosidic oxygen and concomitant hydrogen transfer to that atom from the second sugar in the chain. The next significant fragment is seen at *m*/*z* 631, which is due to cleavage and charge retention on the nonreducing end again, but is now between a Rha(Rib) and Glc residue with retention of the glycosidic oxygen on the reducing end (uncharged) fragment with hydrogen transfer from the Rha to that oxygen. Interestingly, from this point, the principal fragment ions are all due to similar Rha–Glc cleavages along the oligosaccharide backbone, separated by the repeat pentasaccharide unit Rha–Rha(Rib)–Rha–Glc to give *m*/*z* 1363, 2095, 2829, 3562, and 4295. A single (less favored) reducing end fragment, which corresponds to the partial sequence identified in Figs. 3 and 6, is observed at *m*/*z* 1533 in this spectrum, whereby the negative charge would most probably reside on the peptide carboxylate anion. The 6564.6 peak top assignment in the TOF MS calculates for a ^12^C value of 6559.6 for [M − H]^−^ and thus a molecular mass of 6560.6 for the glycopeptide, which is close to the theoretical calculated mass for the new structure.

Interestingly, the apparent anomaly of the MALDI TOF/TOF data on the β-eliminated permethylated samples correlating with a dimethylphosphate-substituted terminal rhamnose moiety is explained by the chemistry of the permethylation reaction and its predictable effect on a cyclic phosphate substitution of this type. The rhamnose cyclophosphate would be subject to nucleophilic attack by hydroxide anion at either of the sugar ring attachment points (C-2 or C-3), which would lead to ring opening to produce a linear phosphate substitution with two hydroxyls for subsequent methylation.

**Figure 9.**
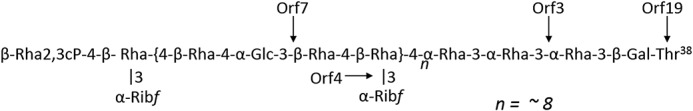
**Deduced overall structure of the glycan attached to SlpA in Ox247 from MS and NMR data.** The structure contains ∼8 copies of a repeat containing Rha, Glc, and Rib*f* attached via a linker containing Rha and Gal and attached via an *O-*linkage to Thr-38 of SlpA. The identity of the capping residue is 2,3 cyclophosphoryl-rhamnose. The genes identified as expressing enzymes mediating steps in the pathway are indicated.

Following the assignment of the overall masses of the oligosaccharide polymers, together with the specific nonreducing terminal mass assignable as corresponding to a possible cyclophospho-deoxyhexose in the corresponding negative ion MALDI TOF/TOF MS/MS data in Fig. 8, the NMR data in Figs. 6*A* and 7 were re-evaluated for evidence of such a moiety. Cyclophosphate substituents have been found on some naturally occurring sugars or have been synthesized, and they appear to be characterized by chemical ^31^P shifts of 18–29 ppm and large coupling constants from 12 to 22 Hz. For example, cyclophosphate at positions 1,2 of mannose demonstrated a chemical shift of 19.7 ppm (45), whereas methyl-l-glycero-α-d-manno-heptopyranoside-6,7-cyclophosphate had a ^31^P chemical shift of 18.3 ppm (46). The 16 ppm chemical shift and 18 Hz coupling observed in the S-layer structure are within these ranges and allow an assignment of a 2,3 cyclophosphate on the terminal rhamnose residue Lys. The overall structure of the glycan attached to SlpA predicted from the NMR and MS interpretations is depicted in Fig. 9.

### Phenotypes of glycosylation-defective mutants

To investigate the functional relevance of S-layer glycosylation, a variety of experiments were carried out on the physiology and behavior of *C. difficile* Ox247 compared with mutants in the glycosylation locus.

**Figure 10.**
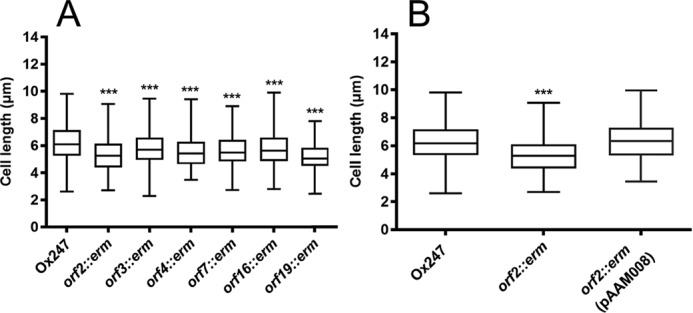
**S-layer glycosylation affects cell length.** Box and whisker plots of cell length of Ox247 and S-layer glycosylation mutants are shown. *A,* Ox247 and mutants. *B,* Ox247, its *orf2* mutant and the *orf2* mutant containing pAAM008 expressing WT *orf2* gene. Strains were grown overnight in BHI broth, and cell lengths were measured from phase-contrast micrographs of 450 cells per strain, using ImageJ. Analysis of variance revealed a statistically significant difference between Ox247 and all mutants (***, *p* < 0.001). Complementation of the mutants in *orf3*, *orf4*, *orf16,* and *orf19* is shown in Fig. S4, *A–D*. The Ox247 and *orf2::erm* data points in *A* are reused in *B*, because the data were acquired from the same experiment.

Ox247 and mutants in *orf2, orf3, orf4, orf7, orf16,* and *orf19* grew normally and did not exhibit any growth defect in liquid media (data not shown). However, we noticed that the cells of every mutant were shorter than the WT (Fig. 10). The average cell of WT Ox247 cells was 6.52 μm, with the mutants having average lengths of 5.34 μm (*orf2*), 5.79 μm (*orf3*), 5.56 μm (*orf4*), 5.68 μm (*orf7*), 5.75 μm (*orf16*), and 5.14 μm (*orf19*). In all mutants (except *orf7* where complementation was not possible), the cell-length defect in the mutant was complemented by a plasmid expressing the WT gene, with a significant (*p* < 0.001) increase in mean cell length occurring (Fig. 10*B* and Fig. S4, *A–D*).

**Figure 11.**
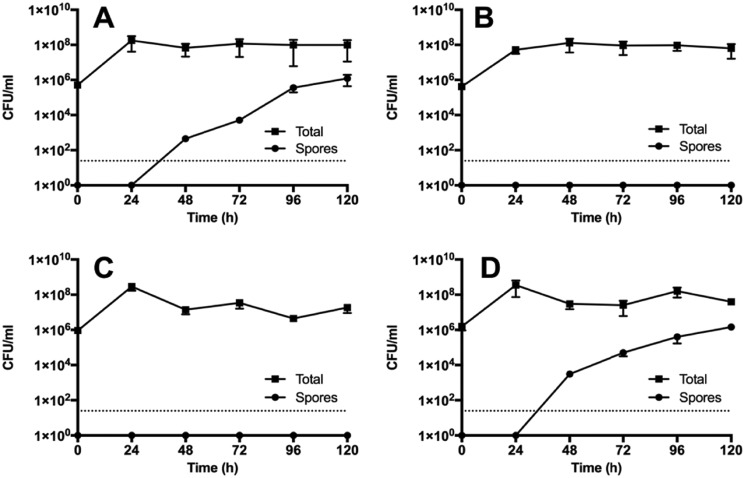
**Sporulation is compromised in glycosylation-deficient mutants.** Overnight cultures of Ox247 (*A*), Ox247 *orf2::erm* (*B*), Ox247 *orf2::erm* (PAAM008) (*C*), and R20291 (*D*) were grown in BHIS broth, and sub-cultured three times. After the final sub-culture (time = 0), samples were taken and assayed for total cells and spores. The R20291 culture was included as a reference to a well-characterized strain. Date are the average ± S.E.

A key phenotype of the *Clostridium* genus is the ability to differentiate to form heat-resistant spores (4). We found that although Ox247 produced spores at a normal rate, the *orf2* and *orf19* mutants were deficient in this process (Fig. 11). After 120 h in liquid culture, Ox247 produced ∼10^6^ spores/ml. In contrast, the Ox247 *orf2* mutant produced no detectable spores at all during this period. Interestingly, the *orf2* mutant containing the WT *orf2* gene behaved similarly to the *orf2* mutant, producing no spores, despite this strain being able to complement the S-layer cell wall protein phenotype as determined by SDS-PAGE (Fig. 2). The sporulation-defective phenotype could not be complemented by plasmids expressing *orf2* from either a constitutive promoter (*P_cwp_*; Fig. 11*C*) or from the anhydrotetracycline-inducible promoter, *P_tet_* (data not shown).

**Figure 12.**
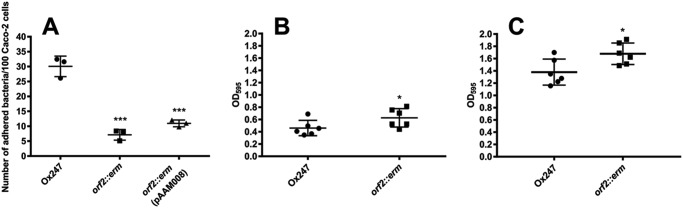
**Adhesion and biofilm formation of *C. difficile* Ox247.** *A,* adhesion to Caco2 cells. Caco2 cell monolayers were infected with *C. difficile* strains with a multiplicity of infection of 5 and incubated for 2 h. After washing, the viable adherent bacteria were measured by growth on BHIS agar. Data are representative of seven individual assays, and the S.E. is shown. ***, *p* < 0.001 when compared with Ox247. *B* and *C,* biofilm formation of Ox247 and the *orf2* mutant was assessed at 24 h (*B*) and 72 h (*C*). *, *p* < 0.05.

Several studies have implicated the S-layer of *C. difficile* to play a key role in its adhesion to host intestinal cells (47, 48). To investigate whether glycosylation of the S-layer affects adhesion to intestinal cells, adhesion of Ox247 and the *orf2* mutant to intestinal Caco-2 cells was compared. As shown in Fig. 12, adhesion was greater in Ox247 compared with its *orf2* mutant cells. A slight restoration in adhesion was observed by the Ox247 *orf2::erm* mutant when complemented with pAAM008 expressing Orf2, but this was not statistically significant. Additionally, we assessed the effect of S-layer glycosylation on the ability to form biofilms. A small but significant increase in biofilm formation was observed in the *orf2* mutant that lacks the glycosylated S-layer (Fig. 12, *B* and *C*).

Several studies have implicated cell-surface components in the cellular behavior of *C. difficile*. For example, overexpression of the cell wall protein CwpV induces aggregation of cells (28), as does increasing the intracellular level of cyclic di-GMP (29). We saw no difference between Ox247 and any of the S-layer glycosylation mutants in aggregation or in flagellar-mediated motility (data not shown).

## Discussion

*C. difficile* is an important bacterial pathogen that presents major healthcare problems around the world (1, 2). Pathogenic strains produce toxins that are the major virulence factors, but cell-surface components are involved in survival *in vivo* and in the transition between a sessile and motile life and between planktonic and biofilm growth. The *C. difficile* S-layer is known to vary between strains, with at least 13 SLCTs identified to date (11, 49). Recently, a promising family of novel antimicrobials, diffocins, have been described that have high activity against *C. difficile* (50). Diffocins, which are derived from phage tails, are highly specific for certain *C. difficile* strains and have been shown to target specifically the *C. difficile* S-layer protein SlpA (49). The discovery that SLCT-11 is glycosylated could have important consequences for the use of diffocins to effectively target the S-layer.

Our study reveals that the S-layer protein of SLCT-11 strains is unique in being a glycoprotein displaying a complex glycan on its surface. The organizational structure of the glycan bears some resemblance to previously described glycans in Gram-positive bacteria (29). MS and NMR analysis revealed the structure of the glycan and showed that it is composed of three domains: (i) a core tetrasaccharide with the sequence -4-α-Rha-3-α-Rha-3-α-Rha-3-β-Gal-; (ii) a repeating pentasaccharide with the sequence -4-β-Rha-4-α-Glc-3-β-Rha-4- (α-Rib-3-)β-Rha-; and (iii) a terminal 2,3 cyclophosphoryl-rhamnose attached to a ribose-branched sub-terminal rhamnose. The overall mass of the glycan identified by MS suggests the presence of at least ∼8 repeat units linked in tandem and attached at the reducing end to the tetrasaccharide core that is *O-*linked to Thr-38 in the SlpA protein (Fig. 9.) Thr-38 is present within the LMW SLP of the S-layer protein, which is located on the external face of the S-layer, whereas the HMW SLP is located on the inner face of the S-layer and is in contact with the underlying polysaccharide–peptidoglycan layer (26).

Our genetic analysis combined with bioinformatics analysis and MS has cast light on the functions of several of the genes in the glycosylation cluster. The *orf2* mutants fail to synthesize a glycan as evidenced by the presence of the 20-kDa form of LMW SLP and the lack of higher molecular weight proteins migrating at ∼45 kDa. Consistent with bioinformatic analysis, Orf2 is predicted to be a undecaprenyl phosphate galactose phosphotransferase that would transfer galactose to undecaprenyl phosphate on the inner face of the cytoplasmic membrane. A similar phenotype is seen with *orf19* mutants, consistent with its role as a ligase, acting on the external face of the membrane to transfer the complete glycan chain to the S-layer at position Thr-38. The *orf3* mutants produce an S-layer with a truncated glycan, containing dHex-Hex as visualized by MS and confirmed by NMR analysis of the WT glycan to be α-Rha-3-β-Gal. Thus, Orf 3 is predicted to be a 1,3-rhamnose rhamnosyltransferase responsible for the addition of 1–3 rhamnose residues to the core reducing end tetrasaccharide glycan. MS analysis of the *orf4* mutant showed glycans that consistently lacked ribose, pointing to this protein being a 1,3-ribosyltransferase, transferring the Ribf to the Rha (Fig. S5). Finally *orf7* mutants produced a truncated oligosaccharide, containing pentose dHex_5_Hex. This suggests that Orf7 may be a glycosyltransferase that attaches glucose to the rhamnose in the repeating structure of the glycan.

The machinery described here for synthesis, assembly, and transport of the glycan and its ligation to the S-layer protein most resembles those described for glycosylation of S-layers from *G. stearothermophilus* and *P. alvei* (27, 29). The glycan is built on the cytoplasmic side of the membrane using endogenous undecaprenyl phosphate as an acceptor, first by addition of a core tetrasaccharide, followed by addition of repeating polysaccharide, and finally a capping moiety that presumably terminates synthesis. The glycan is exported via a two-component ABC transporter and ligated to the protein on the external surface of the cell. Termination of glycan synthesis in the system we describe here appears to involve incorporation of a modified phosphate group on the nonreducing end of the capping group. Phosphate was identified at the nonreducing end of the O9 *O*-polysaccharide of *E. coli* and is required for termination of glycan chain elongation and for subsequent export via the ABC transporter (51). To our knowledge, the presence of phosphate as a constituent of the terminal group of an S-layer glycan has not previously been described, and we speculate that this moiety serves a similar purpose to that in O9a polysaccharide biosynthesis.

*C. difficile* strains can glycosylate other surface structures, notably flagella, by addition of *O*-linked glycans. In some strains, exemplified by strain 630, a phosphorylated *N*-methyl-l-threonine–substituted GlcNAc is present. In ribotype 027 strains, a distinctive modification is found consisting of a novel sulfonated peptidylamido-glycan containing rhamnose residues (52).

Approximately 10–15% of *C. difficile* strains carry SLCT-11, including the genes that compose the glycan biosynthetic machinery (11), and therefore they are expected to display the glycan we describe here. Our previous study showed that other strains of *C. difficile* do not contain a glycosylated S-layer, consistent with their lack of glycosylation machinery (53). These nonglycosylated strains were of ribotypes 001, 012, 010, 016, 017, 027, and 053. However, because the S-layer cassette is thought to recombine by homologous recombination, there is not an absolute association of SLCT with ribotype. The strain we have described here, Ox247, is of ribotype 005 and is within clade 1. However, further analysis of strains within clade 3 has revealed a predominance of strains with SLCT-11,^9^ suggesting that this glycosylated S-layer does to some extent associate more stably with some strains than others.

Analysis of mutants defective in glycosylation of SlpA revealed some interesting phenotypes, suggesting a possible role for the glycan in the fitness and physiology of *C. difficile.* Glycan-negative strains were consistently unable to form spores, suggesting a role for the S-layer in sporulation. Glycan-deficient cells were also shorter than WT cells suggesting a link between cell division or cell architecture and the presence of a complete S-layer. Glycan-deficient cells were also less proficient at adhering to the intestinal Caco2 cell line, suggesting the glycan may be involved in adhesion to gut cells and colonization *in vivo* during infection.

## Experimental procedures

### Bacterial growth and culture

*C. difficile* was routinely cultured in TY broth and on BHIS agar (brain heart infusion agar supplemented with 0.1% l-cysteine and 5 mg/ml yeast extract) at 37 °C without shaking, under anaerobic conditions (80% N_2_, 10% CO_2_, 10% H_2_.) Media were supplemented where appropriate with thiamphenicol (15 μg/ml), cycloserine (250 μg/ml), cefoxitin (81 μg/ml), or lincomycin (20 μg/ml) or erythromycin (5 μg/ml). All *E. coli* strains were grown using Luria-Bertani broth or agar supplemented, where necessary, with 12.5 mg/ml chloramphenicol (Sigma) at 37 °C. Strains and plasmids used in this study are shown in Table S2.

### Genetic techniques

*C. difficile* genomic DNA was isolated as described previously (54) and plasmids were transferred from *E. coli* CA434 to *C. difficile* strains by conjugation as described previously (30). PCRs were performed with KOD Hot Start polymerase (Novagen) using primers as detailed in Table S2. For construction of insertional mutants in *C. difficile,* the ClosTron method was used (30) with modifications. Retargeted plasmids and the relevant oligonucleotides were designed using the algorithm at ClosTron, and the plasmids were synthesized by DNA 2.0. Plasmids were transformed into *E. coli* CA4343 and then transferred by conjugation to *C. difficile* Ox247, where transconjugants were selected on BHIS agar containing 15 μg/ml thiamphenicol (Sigma). Insertion of the intron into the chromosome was by selection of erythromycin (5 μg/ml), and colonies were screened by PCR and DNA sequencing. Mutants were confirmed to have only one insertion by Southern blot analysis (Fig. S3). Mutants were complemented by introducing the WT gene under the control of the constitutive P*_cwp2_* promoter or the anhydrotetracycline-inducible promoter P*_tet_*, as described previously (26).

### S-layer protein purification

S-layer proteins and associated cell wall proteins were prepared by growth of *C. difficile* strains overnight in 50 ml of BHI broth. Cells were centrifuged, washed in 0.1 volume of PBS, and resuspended in 0.01 volume of 0.2 m glycine, pH 2.2. Cells were mixed by rotation for 30 min and centrifuged, and the supernatant containing the SLPs was removed and neutralized by the addition of 2 m Tris base.

### Sporulation and cell-length assays

To enumerate spores, overnight cultures of *C. difficile* grown in BHIS medium supplemented with thiamphenicol, where necessary, were used to inoculate 10 ml of fresh BHIS medium. At intervals, samples of culture were taken and divided. To determine the total number of colony-forming units, one sample was serially diluted and plated on BHIS medium plus 0.1% taurocholate (Sigma). To determine the number of spores, the second sample was heat-killed by incubation for 25 min at 65 °C prior to plating on BHIS medium plus 0.1% taurocholate. The number of spores was subtracted from the total cell counts to give the vegetative cell numbers.

To analyze cell length, *C. difficile* strains were grown in BHIS broth at 37 °C in an anaerobic chamber for 12 h. 0.5 ml of each culture was centrifuged at 4000 × *g* for 2 min. Following gentle resuspension in 150 μl of 1× PBS, 3 μl of culture was placed on top of 1.2% agarose pads and left to dry for 15 min. Slides were visualized using an Eclipse E600 microscope (Nikon), using a Plan Fluor ×100 objective lens, using oil immersion. Images were captured using a Retiga 2000R camera, using the QCapture Pro Software (QImaging), and images were processed using the ImageJ software.

### Biofilm and adhesion assays

#### 

##### Biofilm formation

Single colonies *C. difficile* grown on BHIS agar were used to inoculate pre-reduced BHIS broth. After overnight incubation, 200 μl of each culture at OD_600_ 0.1 was used to inoculate 1.8 ml of pre-reduced BHIS broth on a 24-well plate (Costar).The plates were incubated anaerobically at 37 °C. After 24 or 72 h, the media were gently removed from each well, and the wells were gently washed with 1 ml of pre-reduced PBS. Biofilms were stained with 500 μl of 0.1% filter-sterilized crystal violet for 30 min. 500 μl of 100% methanol was added to solubilize the crystal violet stain followed by incubation for 30 min at room temperature. The OD_595_ of the solution was then measured using a microplate reader (Bio-Rad). Three biological replicates were performed for each strain, and the assay was replicated six times.

##### Adhesion assay

TC7 cells, a sub-clone of the Caco-2 cell line, were maintained using Dulbecco's modified Eagle's medium (DMEM), containing 15% fetal bovine saline (ThermoFisher Scientific), 1% minimum Eagle's medium nonessential amino acids (Life Technologies, Inc.), and 2 mm GlutaMAX (ThermoFisher Scientific). Monolayers were used at 8 days post-confluency, and cells were given nonsupplemented DMEM (containing no fetal bovine serum) 24 h prior to infection. Cells at passage 30–40 were washed with PBS prior to infection. *C. difficile* bacteria were prepared at a multiplicity of infection of 5 in pre-reduced and pre-warmed DMEM. 1 ml of bacterial cells was added to each well of TC7 cells and incubated for 2 h. Bacterial suspensions were then removed, and cells were washed three times with pre-reduced BHIS media. Following vigorous pipetting, TC7 cells with adherent bacteria were removed from the wells, gently vortexed to remove cell clumping, and enumerated on BHIS agar.

### Mass spectrometry

#### 

##### Discovery and glycoproteomic analyses

For discovery and glycoproteomic experiments, the bands of interest from polyacrylamide gels were in-gel digested with trypsin, and the tryptic peptide/glycopeptide digest mixtures, from S-layer *C. difficile* Ox247 both WT and mutants, were analyzed directly by either micro- or nano-LC-ES-MS and MS/MS using Q-TOF mass spectrometers as described previously (31–38): (*a*) a reverse-phase nano-HPLC system ((15 cm × 75 mm inner diameter) PepMap column (LC Packings, Dionex)) connected to a Q-STAR Pulsar (ABI/MDS SCIEX) and/or (*b*) a reverse-phase LC Acquity column (BEH C-18 1 × 50 mm, Waters) connected to a Xevo G2 (Waters) instrument. All interpretations of glycoproteomic data were made manually by visual inspection (36–38). The site of glycan substitution was determined using electron capture dissociation on *C. difficile* WT samples, and both for this work and studies on the *orf3::erm* mutant a Synapt G2-S instrument (Waters) fitted with a nanoAcquity Ultra Performance LC C_18_ column (15 cm length, 75 μm inner diameter) was used. ETD was achieved using glow discharge and *m*-nitrobenzyl alcohol (Sigma) to supercharge the peptides before mass spectrometric detection (39).

##### Sugar composition and linkage

GC-MS compositional analysis of alditol acetates, following TFA hydrolysis, borodeuteride reduction, and re-*N*-acetylation was carried out as described previously (36). Linkage analysis was achieved by the study of partially methylated alditol acetate derivatives as described (40). GC-MS analysis was carried out on a Bruker SCION SQ 456 instrument.

##### Oligosaccharide and glycopeptide mass and sequence analysis

The preparation, purification, and permethylation of glycans β-eliminated from the protein backbone was carried out as described previously (41, 42). MALDI-TOF MS of both free and derivatized oligosaccharides and of free intact glycopeptides was carried out in linear and reflectron modes in both positive and negative ionization, as appropriate, using a 4800 MALDI TOF/TOF (ABI SCIEX) mass spectrometer. For MALDI TOF/TOF MS/MS selected quasi-molecular ions of either permethylated or underivatized species observed in the MS spectra were subjected to collision-induced dissociation, at a collision energy of 1 kV with argon as the collision gas (41, 42).

### Large-scale glycan purification for NMR analysis

*C. difficile* Ox247 was grown in BHIS broth (6× 1 liter) for 16 h at 37 °C in an anaerobic chamber. Bacterial cells were harvested, and the cell pellets were resuspended in 0.2 m glycine buffer and incubated at room temperature for 20 min with gentle mixing. Cells were removed by centrifugation for 15 min in an Eppendorf microcentrifuge, and glycine supernatants were neutralized with 1 m Tris. Following dialysis against dH_2_O for 48 h, the S-layer protein extract was digested with proteinase K (Sigma) in 10 mm NaPO_4_, pH 8.0, at 37 °C for 48 h (5:1 ratio S-layer protein/proteinase K). The digested samples were then subjected to Amicon filtration (3000-Da cutoff), and the retentate was lyophilized. The lyophilized sample was resuspended in dH_2_O and further purified as described below.

Polysaccharides were purified by anion-exchange chromatography on Hitrap Q column in a linear gradient from water to 1 m NaCl over 1 h with UV detection at 220 nm and a spot test on TLC plate with development by dipping in 5% H_2_SO_4_ in ethanol and heating with a heat gun until brown spots became visible, and products were desalted by gel chromatography. Samples were desalted on Sephadex G-15 column (1.6 × 60 cm) in 1% AcOH with refractive index detector.

### NMR spectroscopy

NMR experiments were carried out on a Bruker AVANCE III 600 MHz (^1^H) spectrometer with a 5-mm Z-gradient probe with acetone internal reference (2.225 ppm for ^1^H and 31.45 ppm for ^13^C) using standard pulse sequences cosygpprqf (gCOSY), mlevphpr (TOCSY, mixing time 120 ms), roesyphpr (ROESY, mixing time 500 ms), hsqcedetgp (HSQC), hsqcetgpml (HSQC-TOCSY, 80 ms TOCSY delay), and hmbcgplpndqf (HMBC, 100-ms long-range transfer delay). Resolution was kept <3 Hz/point in F2 in proton–proton correlations and <5 Hz/pt in F2 of H–C correlations. The spectra were processed and analyzed using the Bruker Topspin 2.1 program.

## Author contributions

E. R., L. B., M. P., A. A., S. M. L., and A. D. data curation; E. R., M. P., A. A., E. V., H. M., S. M. L., A. D., and N. F. F. formal analysis; E. R., L. B., M. P., A. A., E. V., H. M., A. D., and N. F. F. investigation; E. R., L. B., M. P., E. V., H. M., and S. M. L. methodology; E. R., L. B., E. V., H. M., B. W., S. M. L., A. D., and N. F. F. writing-original draft; H. M., S. M. L., A. D., and N. F. F. supervision; H. M., B. W., S. M. L., A. D., and N. F. F. writing-review and editing; B. W., S. M. L., A. D., and N. F. F. conceptualization; B. W., S. M. L., A. D., and N. F. F. funding acquisition; B. W., A. D., and N. F. F. project administration.

## Supplementary Material

Supporting Information

## Footnotes

9 A. Shaw and B. Wren, manuscript in preparation.

## Footnotes

8 The abbreviations used are:

CDI: *C. difficile* infection
SLP: S-layer protein
ETD: electron transfer dissociation
LMW: low-molecular-weight
HMW: high-molecular-weight
SLC: S-layer cassette
SLCT: SLC type
dH_2_O: distilled H_2_O
DMEM: Dulbecco's modified Eagle's medium
HSQC: heteronuclear single quantum coherence
TOCSY: total correlation spectroscopy
ROESY: rotating frame nuclear Overhauser enhancement spectroscopy
COSY: two-dimensional correlated spectroscopy
HMBC: heteronuclear multiple bond correlation
Hex: hexose
dHex: deoxyhexose
Pent: pentose.

## References

[1] Smits W. K., Lyras D., Lacy D. B., Wilcox M. H., and Kuijper E. J. (2016) *Clostridium difficile* infection. Nat. Rev. Dis. Primers 2, 16021 10.1038/nrdp.2016.21 27158839 PMC5453186

[2] Abt M. C., McKenney P. T., and Pamer E. G. (2016) *Clostridium difficile* colitis: pathogenesis and host defence. Nat. Rev. Microbiol. 14, 609–620 10.1038/nrmicro.2016.108 27573580 PMC5109054

[3] Jank T., and Aktories K. (2008) Structure and mode of action of clostridial glucosylating toxins: the ABCD model. Trends Microbiol. 16, 222–229 10.1016/j.tim.2008.01.011 18394902

[4] Deakin L. J., Clare S., Fagan R. P., Dawson L. F., Pickard D. J., West M. R., Wren B. W., Fairweather N. F., Dougan G., and Lawley T. D. (2012) The *Clostridium difficile spo0A* gene is a persistence and transmission factor. Infect. Immun. 80, 2704–2711 10.1128/IAI.00147-12 22615253 PMC3434595

[5] Lawley T. D., Clare S., Walker A. W., Goulding D., Stabler R. A., Croucher N., Mastroeni P., Scott P., Raisen C., Mottram L., Fairweather N. F., Wren B. W., Parkhill J., and Dougan G. (2009) Antibiotic treatment of *Clostridium difficile* carrier mice triggers a supershedder state, spore-mediated transmission, and severe disease in immunocompromised hosts. Infect. Immun. 77, 3661–3669 10.1128/IAI.00558-09 19564382 PMC2737984

[6] Chang J. Y., Antonopoulos D. A., Kalra A., Tonelli A., Khalife W. T., Schmidt T. M., and Young V. B. (2008) Decreased diversity of the fecal Microbiome in recurrent *Clostridium difficile*-associated diarrhea. J. Infect. Dis. 197, 435–438 10.1086/525047 18199029

[7] Seekatz A. M., Rao K., Santhosh K., and Young V. B. (2016) Dynamics of the fecal microbiome in patients with recurrent and nonrecurrent *Clostridium difficile* infection. Genome Med. 8, 47 10.1186/s13073-016-0298-8 27121861 PMC4847246

[8] Fagan R. P., and Fairweather N. F. (2014) Biogenesis and functions of bacterial S-layers. Nat. Rev. Microbiol. 12, 211–222 10.1038/nrmicro3213 24509785

[9] Kirk J. A., Banerji O., and Fagan R. P. (2017) Characteristics of the *Clostridium difficile* cell envelope and its importance in therapeutics. Microb. Biotechnol. 10, 76–90 27311697 10.1111/1751-7915.12372 PMC5270738

[10] Calabi E., Ward S., Wren B., Paxton T., Panico M., Morris H., Dell A., Dougan G., and Fairweather N. (2001) Molecular characterization of the surface layer proteins from *Clostridium difficile*. Mol. Microbiol. 40, 1187–1199 10.1046/j.1365-2958.2001.02461.x 11401722

[11] Dingle K. E., Didelot X., Ansari M. A., Eyre D. W., Vaughan A., Griffiths D., Ip C. L., Batty E. M., Golubchik T., Bowden R., Jolley K. A., Hood D. W., Fawley W. N., Walker A. S., Peto T. E., et al (2013) Recombinational switching of the *Clostridium difficile* S-layer and a novel glycosylation gene cluster revealed by large-scale whole-genome sequencing. J. Infect. Dis. 207, 675–686 10.1093/infdis/jis734 23204167 PMC3549603

[12] Sleytr U. B., and Messner P. (1988) Crystalline surface layers in procaryotes. J. Bacteriol. 170, 2891–2897 10.1128/jb.170.7.2891-2897.1988 3290191 PMC211226

[13] Sára M., and Sleytr U. B. (2000) S-layer proteins. J. Bacteriol. 182, 859–868 10.1128/JB.182.4.859-868.2000 10648507 PMC94357

[14] Beveridge T. J., Pouwels P. H., Sára M., Kotiranta A., Lounatmaa K., Kari K., Kerosuo E., Haapasalo M., Egelseer E. M., Schocher I., Sleytr U. B., Morelli L., Callegari M. L., Nomellini J. F., Bingle W. H., et al (1997) Functions of S-layers. FEMS Microbiol. Rev. 20, 99–149 10.1016/S0168-6445(97)00043-0,10.1111/j.1574-6976.1997.tb00305.x 9276929

[15] Ryan A., Lynch M., Smith S. M., Amu S., Nel H. J., McCoy C. E., Dowling J. K., Draper E., O'Reilly V., McCarthy C., O'Brien J., Ní Eidhin D., O'Connell M. J., Keogh B., Morton C. O., et al (2011) A role for TLR4 in *Clostridium difficile* infection and the recognition of surface layer proteins. PLoS Pathog. 7, e1002076 10.1371/journal.ppat.1002076 21738466 PMC3128122

[16] Kern J., and Schneewind O. (2010) BslA, the S-layer adhesin of B. anthracis, is a virulence factor for anthrax pathogenesis. Mol. Microbiol. 75, 324–332 10.1111/j.1365-2958.2009.06958.x 19906175 PMC2828814

[17] Tarlovsky Y., Fabian M., Solomaha E., Honsa E., Olson J. S., and Maresso A. W. (2010) A *Bacillus anthracis* S-layer homology protein that binds heme and mediates heme delivery to IsdC. J. Bacteriol. 192, 3503–3511 10.1128/JB.00054-10 20435727 PMC2897652

[18] Baranova E., Fronzes R., Garcia-Pino A., Van Gerven N., Papapostolou D., Péhau-Arnaudet G., Pardon E., Steyaert J., Howorka S., and Remaut H. (2012) SbsB structure and lattice reconstruction unveil Ca^2+^ triggered S-layer assembly. Nature 487, 119–122 10.1038/nature11155 22722836

[19] Arbing M. A., Chan S., Shin A., Phan T., Ahn C. J., Rohlin L., and Gunsalus R. P. (2012) Structure of the surface layer of the methanogenic archaean *Methanosarcina acetivorans*. Proc. Natl. Acad. Sci. U.S.A. 109, 11812–11817 10.1073/pnas.1120595109 22753492 PMC3406845

[20] Jing H., Takagi J., Liu J. H., Lindgren S., Zhang R. G., Joachimiak A., Wang J. H., and Springer T. A. (2002) Archaeal surface layer proteins contain β propeller, PKD, and β helix domains and are related to metazoan cell surface proteins. Structure 10, 1453–1464 10.1016/S0969-2126(02)00840-7 12377130

[21] Anderson V. J., Kern J. W., McCool J. W., Schneewind O., and Missiakas D. (2011) The SLH-domain protein BslO is a determinant of *Bacillus anthracis* chain length. Mol. Microbiol. 81, 192–205 10.1111/j.1365-2958.2011.07688.x 21585566 PMC3124567

[22] Kern J., Wilton R., Zhang R., Binkowski T. A., Joachimiak A., and Schneewind O. (2011) Structure of the SLH domains from *Bacillus anthracis* surface array protein. J. Biol. Chem. 286, 26042–26049 10.1074/jbc.M111.248070 21572039 PMC3138252

[23] Fagan R. P., Albesa-Jové D., Qazi O., Svergun D. I., Brown K. A., and Fairweather N. F. (2009) Structural insights into the molecular organization of the S-layer from *Clostridium difficile*. Mol. Microbiol. 71, 1308–1322 10.1111/j.1365-2958.2009.06603.x 19183279

[24] Bharat T. A. M., Kureisaite-Ciziene D., Hardy G. G., Yu E. W., Devant J. M., Hagen W. J. H., Brun Y. V., Briggs J. A. G., and Löwe J. (2017) Structure of the hexagonal surface layer on *Caulobacter crescentus* cells. Nat. Microbiol. 2, 17059 10.1038/nmicrobiol.2017.59 28418382 PMC5699643

[25] Mesnage S., Tosi-Couture E., Mock M., and Fouet A. (1999) The S-layer homology domain as a means for anchoring heterologous proteins on the cell surface of *Bacillus anthracis*. J. Appl. Microbiol. 87, 256–260 10.1046/j.1365-2672.1999.00880.x 10475961

[26] Willing S. E., Candela T., Shaw H. A., Seager Z., Mesnage S., Fagan R. P., and Fairweather N. F. (2015) *Clostridium difficile* surface proteins are anchored to the cell wall using CWB2 motifs that recognise the anionic polymer PSII. Mol. Microbiol. 96, 596–608 10.1111/mmi.12958 25649385 PMC4973711

[27] Ristl R., Steiner K., Zarschler K., Zayni S., Messner P., and Schäffer C. (2011) The S-layer glycome-adding to the sugar coat of bacteria. Int. J. Microbiol. 2011, 127870 20871840 10.1155/2011/127870 PMC2943079

[28] Abu-Qarn M., Eichler J., and Sharon N. (2008) Not just for Eukarya anymore: protein glycosylation in bacteria and archaea. Curr. Opin. Struct. Biol. 18, 544–550 10.1016/j.sbi.2008.06.010 18694827

[29] Schäffer C., and Messner P. (2017) Emerging facets of prokaryotic glycosylation. FEMS Microbiol. Rev. 41, 49–91 27566466 10.1093/femsre/fuw036 PMC5266552

[30] Heap J. T., Pennington O. J., Cartman S. T., Carter G. P., and Minton N. P. (2007) The ClosTron: a universal gene knock-out system for the genus *Clostridium*. J. Microbiol. Methods 70, 452–464 10.1016/j.mimet.2007.05.021 17658189

[31] Dell A., and Morris H. R. (2001) Glycoprotein structure determination by mass spectrometry. Science 291, 2351–2356 10.1126/science.1058890 11269315

[32] Moody A. M., North S. J., Reinhold B., Van Dyken S. J., Rogers M. E., Panico M., Dell A., Morris H. R., Marth J. D., and Reinherz E. L. (2003) Sialic acid capping of CD8β core 1-*O*-glycans controls thymocyte-major histocompatibility complex class I interaction. J. Biol. Chem. 278, 7240–7246 10.1074/jbc.M210468200 12459555

[33] van Der Wel H., Morris H. R., Panico M., Paxton T., North S. J., Dell A., Thomson J. M., and West C. M. (2001) A non-Golgi α1,2-fucosyltransferase that modifies Skp1 in the cytoplasm of *Dictyostelium*. J. Biol. Chem. 276, 33952–33963 10.1074/jbc.M102555200 11423539

[34] Morris H. R., Chalabi S., Panico M., Sutton-Smith M., Clark G. F., Goldberg D., and Dell A. (2007) Glycoproteomics: past, present and future. Int. J. Mass Spectrom. 259, 16–31 10.1016/j.ijms.2006.09.002

[35] Morris H. R., Paxton T., Dell A., Langhorne J., Berg M., Bordoli R. S., Hoyes J., and Bateman R. H. (1996) High sensitivity collisionally-activated decomposition tandem mass spectrometry on a novel quadrupole/orthogonal-acceleration time-of-flight mass spectrometer. Rapid Commun. Mass Spectrom. 10, 889–896 10.1002/(SICI)1097-0231(19960610)10:8%3C889::AID-RCM615%3E3.0.CO%3B2-F 8777321

[36] Bouché L., Panico M., Hitchen P., Binet D., Sastre F., Faulds-Pain A., Valiente E., Vinogradov E., Aubry A., Fulton K., Twine S., Logan S. M., Wren B. W., Dell A., and Morris H. R. (2016) The type B flagellin of hypervirulent *Clostridium difficile* is modified with novel sulfonated peptidylamido-glycans. J. Biol. Chem. 291, 25439–25449 10.1074/jbc.M116.749481 27758867 PMC5207245

[37] Panico M., Bouché L., Binet D., O'Connor M. J., Rahman D., Pang P. C., Canis K., North S. J., Desrosiers R. C., Chertova E., Keele B. F., Bess J. W. Jr., Lifson J. D., Haslam S. M., Dell A., and Morris H. R. (2016) Mapping the complete glycoproteome of virion-derived HIV-1 gp120 provides insights into broadly neutralizing antibody binding. Sci. Rep. 6, 32956 10.1038/srep32956 27604319 PMC5015092

[38] Morris H. R., Paxton T., Panico M., McDowell R., and Dell A. (1997) A novel geometry mass spectrometer, the Q-TOF, for low-femtomole/attomole-range biopolymer sequencing. J. Protein Chem. 16, 469–479 10.1023/A:1026309410737 9246631

[39] Iavarone A. T., and Williams E. R. (2002) Supercharging in electrospray ionization: effects on signal and charge. Int. J. Mass Spectrom. 219, 63–72 10.1016/S1387-3806(02)00587-0

[40] Sweet D. P., Shapiro R. H., and Albersheim P. (1974) The mass spectral fragmentation of partially ethylated alditol acetates, a derivative used in determining the glycosyl linkage composition of polysaccharides. Biomed. Mass. Spectrom. 1, 263–268 10.1002/bms.1200010410 4441621

[41] Jang-Lee J., North S. J., Sutton-Smith M., Goldberg D., Panico M., Morris H., Haslam S., and Dell A. (2006) Glycomic profiling of cells and tissues by mass spectrometry: fingerprinting and sequencing methodologies. Methods Enzymol. 415, 59–86 10.1016/S0076-6879(06)15005-3 17116468

[42] Canis K., McKinnon T. A., Nowak A., Haslam S. M., Panico M., Morris H. R., Laffan M. A., and Dell A. (2012) Mapping the *N*-glycome of human von Willebrand factor. Biochem. J. 447, 217–228 10.1042/BJ20120810 22849435

[43] Fagan R., and Fairweather N. (2010) Dissecting the cell surface. Methods Mol. Biol. 646, 117–134 10.1007/978-1-60327-365-7_8 20597006

[44] Ganeshapillai J., Vinogradov E., Rousseau J., Weese J. S., and Monteiro M. A. (2008) *Clostridium difficile* cell-surface polysaccharides composed of pentaglycosyl and hexaglycosyl phosphate repeating units. Carbohydr. Res. 343, 703–710 10.1016/j.carres.2008.01.002 18237724

[45] Sernee M. F., Ralton J. E., Dinev Z., Khairallah G. N., O'Hair R. A., Williams S. J., and McConville M. J. (2006) *Leishmania* β-1,2-mannan is assembled on a mannose-cyclic phosphate primer. Proc. Natl. Acad. Sci. U.S.A. 103, 9458–9463 10.1073/pnas.0603539103 16766650 PMC1480429

[46] Stewart A., Bernlind C., Martin A., Oscarson S., Richards J. C., and Schweda E. K. H. (1998) Studies of alkaline mediated phosphate migration in synthetic phosphoethanolamine l-glycero-d-manno-heptoside derivatives. Carbohydr. Res. 313, 193–202 10.1016/S0008-6215(98)00271-7

[47] Calabi E., Calabi F., Phillips A. D., and Fairweather N. (2002) Binding of *Clostridium difficile* surface layer proteins to gastrointestinal tissues. Infect. Immun. 70, 5770–5778 10.1128/IAI.70.10.5770-5778.2002 12228307 PMC128314

[48] Merrigan M. M., Venugopal A., Roxas J. L., Anwar F., Mallozzi M. J., Roxas B. A., Gerding D. N., Viswanathan V. K., and Vedantam G. (2013) Surface-layer protein A (SlpA) is a major contributor to host-cell adherence of *Clostridium difficile*. PLoS ONE 8, e78404 10.1371/journal.pone.0078404 24265687 PMC3827033

[49] Kirk J. A., Gebhart D., Buckley A. M., Lok S., Scholl D., Douce G. R., Govoni G. R., and Fagan R. P. (2017) New class of precision antimicrobials redefines role of *Clostridium difficile* S-layer in virulence and viability. Sci. Transl. Med. 6, aah6813 10.1126/scitranslmed.aah6813 28878013 PMC5603275

[50] Gebhart D., Williams S. R., Bishop-Lilly K. A., Govoni G. R., Willner K. M., Butani A., Sozhamannan S., Martin D., Fortier L.-C., and Scholl D. (2012) Novel high-molecular-weight, R-type bacteriocins of *Clostridium difficile*. J. Bacteriol. 194, 6240–6247 10.1128/JB.01272-12 22984261 PMC3486368

[51] Clarke B. R., Richards M. R., Greenfield L. K., Hou D., Lowary T. L., and Whitfield C. (2011) *In vitro* reconstruction of the chain termination reaction in biosynthesis of the *Escherichia coli* O9a *O*-polysaccharide: the chain-length regulator, WbdD, catalyzes the addition of methyl phosphate to the non-reducing terminus of the growing glycan. J. Biol. Chem. 286, 41391–41401 10.1074/jbc.M111.295857 21990359 PMC3308851

[52] Faulds-Pain A., Twine S. M., Vinogradov E., Strong P. C., Dell A., Buckley A. M., Douce G. R., Valiente E., Logan S. M., and Wren B. W. (2014) The post-translational modification of the *Clostridium difficile* flagellin affects motility, cell surface properties and virulence. Mol. Microbiol. 94, 272–289 10.1111/mmi.12755 25135277 PMC4441256

[53] Qazi O., Hitchen P., Tissot B., Panico M., Morris H. R., Dell A., and Fairweather N. (2009) Mass spectrometric analysis of the S-layer proteins from *Clostridium difficile* demonstrates the absence of glycosylation. J. Mass Spectrom. 44, 368–374 10.1002/jms.1514 18932172

[54] Emerson J. E., Reynolds C. B., Fagan R. P., Shaw H. A., Goulding D., and Fairweather N. F. (2009) A novel genetic switch controls phase variable expression of CwpV, a *Clostridium difficile* cell wall protein. Mol. Microbiol. 74, 541–556 10.1111/j.1365-2958.2009.06812.x 19656296 PMC2784873

