# Supplementary material for: The S-layer protein of a Clostridium difficile SLCT-11 strain displays a complex glycan required for normal cell growth and morphology
Source: J Biol Chem. 2018 Oct 1;293(47):18123–37. doi: 10.1074/jbc.RA118.004530 (PMC6254364; doi:10.1074/jbc.RA118.004530)
Supplement: Supporting Information [file supp_293_47_18123__index.html]

The S-layer protein of a Clostridium difficile SLCT-11 strain displays a complex glycan required for normal cell growth and morphology — S-layer glycosylation in Clostridium difficile — The S-layer protein of a Clostridium difficile SLCT-11 strain displays a complex glycan required for normal cell growth and morphology — S-layer glycosylation in Clostridium difficile — The S-layer protein of a Clostridium difficile SLCT-11 strain displays a complex glycan required for normal cell growth and morphology — S-layer glycosylation in Clostridium difficile — The S-layer protein of a Clostridium difficile SLCT-11 strain displays a complex glycan required for normal cell growth and morphology — S-layer glycosylation in Clostridium difficile — The S-layer protein of a Clostridium difficile SLCT-11 strain displays a complex glycan required for normal cell growth and morphology — S-layer glycosylation in Clostridium difficile — The S-layer protein of a Clostridium difficile SLCT-11 strain displays a complex glycan required for normal cell growth and morphology — S-layer glycosylation in Clostridium difficile — The S-layer protein of a Clostridium difficile SLCT-11 strain displays a complex glycan required for normal cell growth and morphology — S-layer glycosylation in Clostridium difficile — The S-layer protein of a Clostridium difficile SLCT-11 strain displays a complex glycan required for normal cell growth and morphology — S-layer glycosylation in Clostridium difficile — Supporting Information 

# The S-layer protein of a *Clostridium difficile* SLCT-11 strain displays a complex glycan required for normal cell growth and morphology

## Supporting Information

- Supporting Information (to be published online) - Supporting Information
