# Supplementary material for: The S-layer protein of a Clostridium difficile SLCT-11 strain displays a complex glycan required for normal cell growth and morphology
Source: J Biol Chem. 2018 Oct 1;293(47):18123–37. doi: 10.1074/jbc.RA118.004530 (PMC6254364; doi:10.1074/jbc.RA118.004530)
Supplement: Supporting Information [file supp_RA118.004530_138905_1_supp_211971_pfx22m.pdf]

The S-layer protein of a *Clostridium difficile* SLCT-11 strain displays a complex glycan required for normal cell growth and morphology

Emma Richards[a1], Laura Bouché[a1], Maria Panico[1,] Ana Arbeloa[1], Evgeny Vinogradov[2,] Howard Morris[1,3], Brendan Wren[4], Susan M. Logan[2] Anne Dell[1*] and Neil F. Fairweather[1*]

Supporting Information

Table S1 Predicted functions of genes present in the S-layer glycosylation cluster

Table S2A Bacterial strains used in this study

Table S2B Plasmids used in this study

Table S2C Oligonucleotides used in this study

Figure S1 MS data of Ox247 *orf3* mutant

Figure S2 The GC-EIMS total ion current chromatograms for the alditol acetate derivatives of hydrolysed sugars from Ox247 LMW SLP

Figure S3 Southern blot analysis of Ox247 mutants

Figure S4 Complementation of cell length defects

Figure S5 Summed MS data acquired at 40.2 min in the *online* nanoLC-ES-MS of a tryptic digest of the band at 20 kDa of Ox247 *orf4*::*erm* mutant.

Table S1. Predicted functions of genes present in the S-layer glycosylation cluster

Gene	Closest species match [1]	Identity (%)	Accession Number	Conserved Domains Identified[2]	Putative Function	Homolog in *Geobacillus stearothermophilus* S-layer glycosylation system [3]
orf1	-	-	-	none	-	-
orf2	*Firmicutes bacterium* CAG:424	57	CDC47796.1	WcaJ_sugtrans	Undecaprenyl-phosphate glucose phosphotransferase	WsaP
orf3	*Methanobacterium*	58	WP_048190924.1	GT_2_like_c	Glycosyltransferase	-
orf4	*Planctomycetes bacterium*	44	OHB99936.1	PKK12324<br>PT_UbiA_2	Phosphoribose diphosphate--decaprenyl-phosphate phosphoribosyltransferase	-
orf5	*Paenibacillus macerans*	30	OMG48725.1	YfhO<br>Glucos_Trans_II	Glycosyltransferase	-
orf6	*Methanoculleus sediminis*	56	WP_082122685.1	Glyco_tran_WbsX	Glycosyltransferase	-
orf7	*Paenibacillus polymyxa*	43	WP_023990592.1	Glyco_tran_WbsX	Glycosyltransferase	-
orf8	*Marichromatium gracile*	50	WP_062274959	GT8_Glycogenin<br>Glyco_Transf_8	Glycosyltransferase	-
orf9	*Clostridium beijerinckii*	37	WP_077854956.1	Glyco_tranf_GTA_type	Glycosyltransferase	-
orf10	*Clostridium butyricum*	71	WP 035762026	AdoMet_MTase	SAM methyltransferase<br>Glycosyltransferase	-
orf11	*Clostridium beijerinckii*	65	WP_077868321	ABC_Kps_Wzt<br>Wzt_C-like	ABC transporter; ATP binding protein	Wzt
orf12	*Romboutsia maritimum*	79	WP_095406846.1	TagG<br>ABC2_membrane	ABC superfamily transporter: permease	Wzm
orf13	*Romboutsia maritimum*	73	ZP_08166203.1	RfbB	dTDP-glucose 4,6-dehydratase	RmlB
orf14	*Clostridium perfringens*	72	ZP_02863393.1	dTDP_sugar_isom	dTDP-4-dehydrorhamnose 3,5-epimerase	RmlC
orf15	*Paenibacillus sp. NAIST15-1*	49	GAV12381.1	none	ABC transporter related protein	
orf16	*Multispecies Clostridiales*	98	ZP_08166199.1	rmlA<br>G1P_TT_shor	Glucose-1-phosphate thymidylyltransferase	RmlA

orf17	*Candidatus Dorea massiliensis*	75	WP_053832674.1	RfbD	dTDP-4-dehydrorhamnose reductase	RmlD
orf18	*Romboutsia maritimum*	65	WP_095406851.1	BcsA Glyco_tranf_GTA_type	glycosyltransferase	-
orf19	*Romboutsia lituseburensis*	47	YP_004586423.1	sorted_by_XrtN	O-antigen polymerase/ligase	WsaB

[1] ORFs were analysed using BLASTP. To aid interpretation, *Clostridium difficile* was removed from the search database
[2] Top 1 or 2 hits from a Conserved Domain Database search
[3] Novotny et al (2004) Microbiology 150, 953–965

Table S2. Bacterial strains, plasmids and oligonucleotides used in this study

A. Strains

Strain	Characteristics and source
C. difficile Ox247	Ribotype 005, SLCT-11; Kate Dingle (1)
C. difficile Ox247 *orf2::erm*	Insertional mutant in *orf2*; this study
C. difficile Ox247 *orf3::erm*	Insertional mutant in *orf3*; this study
C. difficile Ox247 *orf4::erm*	Insertional mutant in *orf4*; this study
C. difficile Ox247 *orf7::erm*	Insertional mutant in *orf7*; this study
C. difficile Ox247 *orf16::erm*	Insertional mutant in *orf16*; this study
C. difficile Ox247 *orf19::erm*	Insertional mutant in *orf19*; this study
C. difficile 630	Ribotype 012, Peter Mullany (2)
C. difficile R20291	Ribotype 027; SLCT-4; Brendan Wren (3)

B. Plasmids

Plasmid	Description
pMTL007C-E5-orf2	ClosTron plasmid retargeting *orf3* in Ox247 glycosylation cluster
pMTL007C-E5-orf3	ClosTron plasmid retargeting *orf3* in Ox247 glycosylation cluster
pMTL007C-E5-orf4	ClosTron plasmid retargeting *orf4* in Ox247 glycosylation cluster
pMTL007C-E5-orf7	ClosTron plasmid retargeting *orf7* in Ox247 glycosylation cluster
pMTL007C-E5-orf16	ClosTron plasmid retargeting *orf16* in Ox247 glycosylation cluster
pMTL007C-E5-orf19	ClosTron plasmid retargeting *orf19* in Ox247 glycosylation cluster
pAAM008	pRPF185 with *orf2* cloned into BamHI and SacI sites
pAAM009	pRPF185 with *orf19* cloned into BamHI and SacI sites
pAAM010	pRPF144 with *orf2* cloned into BamHI and SacI sites
pAAM011	pRPF144 with *orf19* cloned into BamHI and SacI sites
pEJR003	pRPF185 with *orf3* cloned to BamHI and SacI sites
pEJR004	pRPF144 with *orf3* cloned to BamHI and SacI sites
pEJR006	pRPF144 with *orf4* cloned into BamHI and SacI sites
pEJR007	pRPF185 with *orf4* cloned into BamHI and SacI sites
pEJR008	pRPF144 with *orf16* cloned into BamHI and SacI sites
pEJR009	pRPF185 with *orf16* cloned into BamHI and SacI sites

Figure S1 A

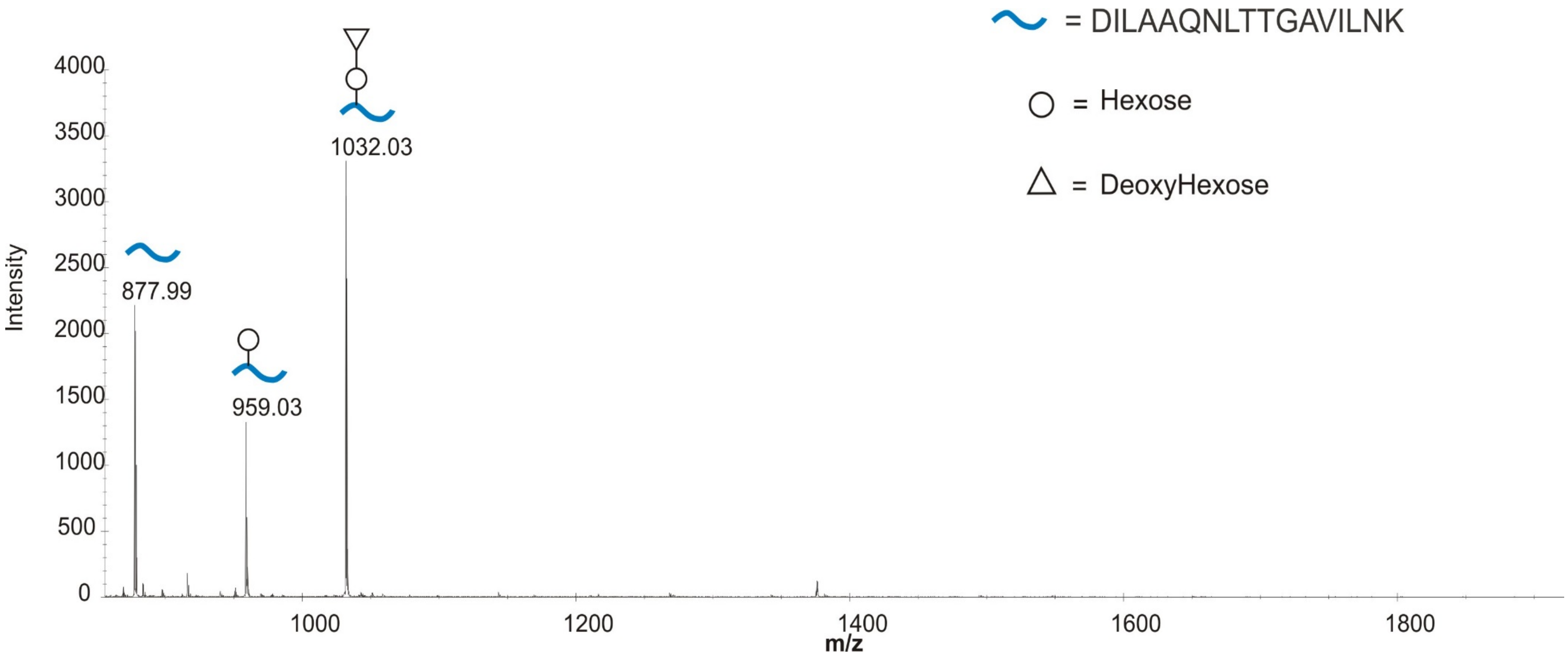

Fig. S1 A. Summed MS data acquired at 44.0 min in the *online* nanoLC-ES-MS of a tryptic digest of the 20 kDa band of the Ox247 *orf3::erm* mutant. Expanded middle mass region shows doubly-charged peaks that corresponds to glycans attached to the LMW SLP.

Figure S1 B

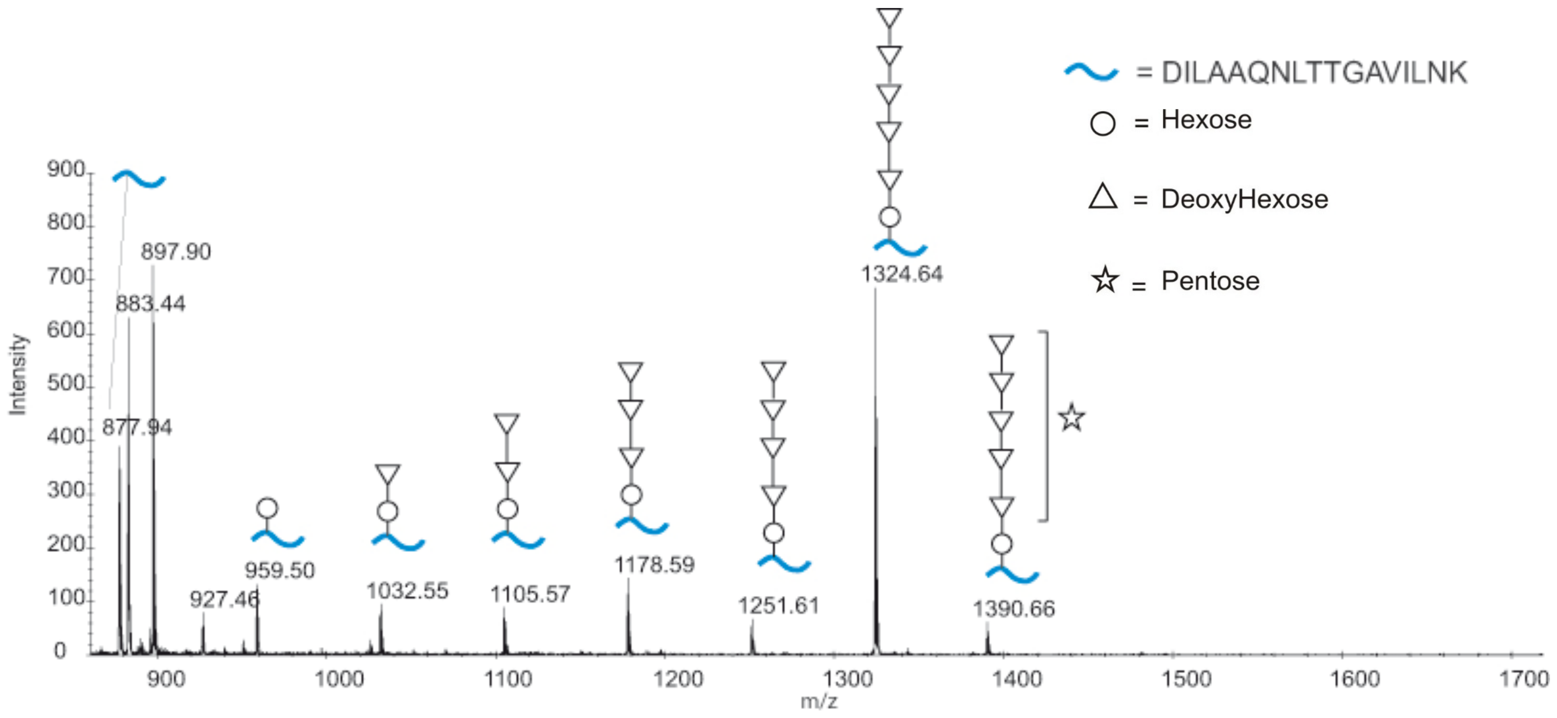

Fig S1 B. Summed MS data acquired at 40.6 min in the *online* nanoLC-ES-MS of a tryptic digest digest of the band at 25 kDa of the Ox247 *orf7::erm* mutant. Expanded middle mass region shows doubly-charged peaks that corresponds to glycan ranging from a single hexose up to Pent$deoxyHex_5$Hex.

Figure S2

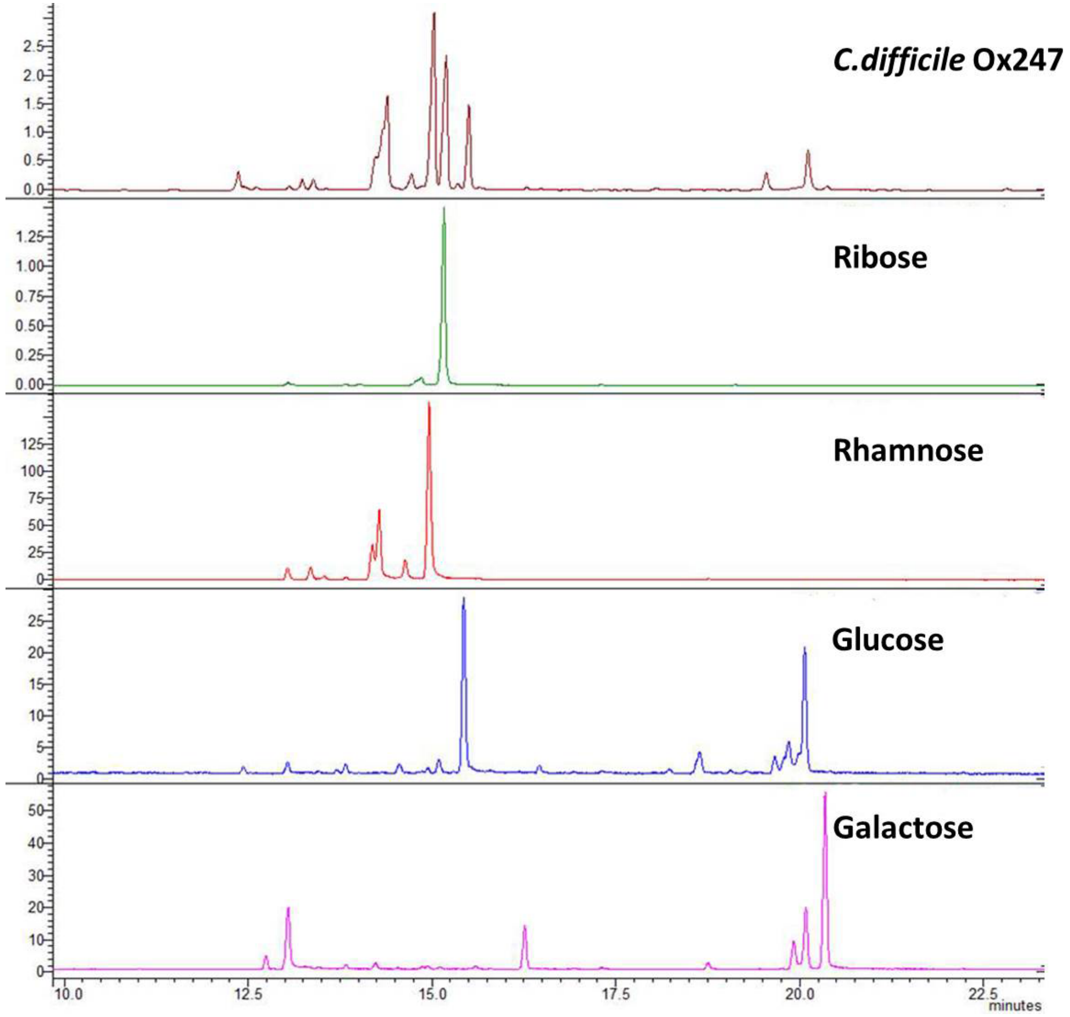

Fig S2. The GC-EIMS total ion current chromatograms for the alditol acetate derivatives of hydrolysed sugars from the LMW SLP WT Ox247 (top trace) compared to individual sugar standards taken through the same protocol (40). For interpretation, see the text.

Figure S3 Southern blot analysis of Ox247 mutants

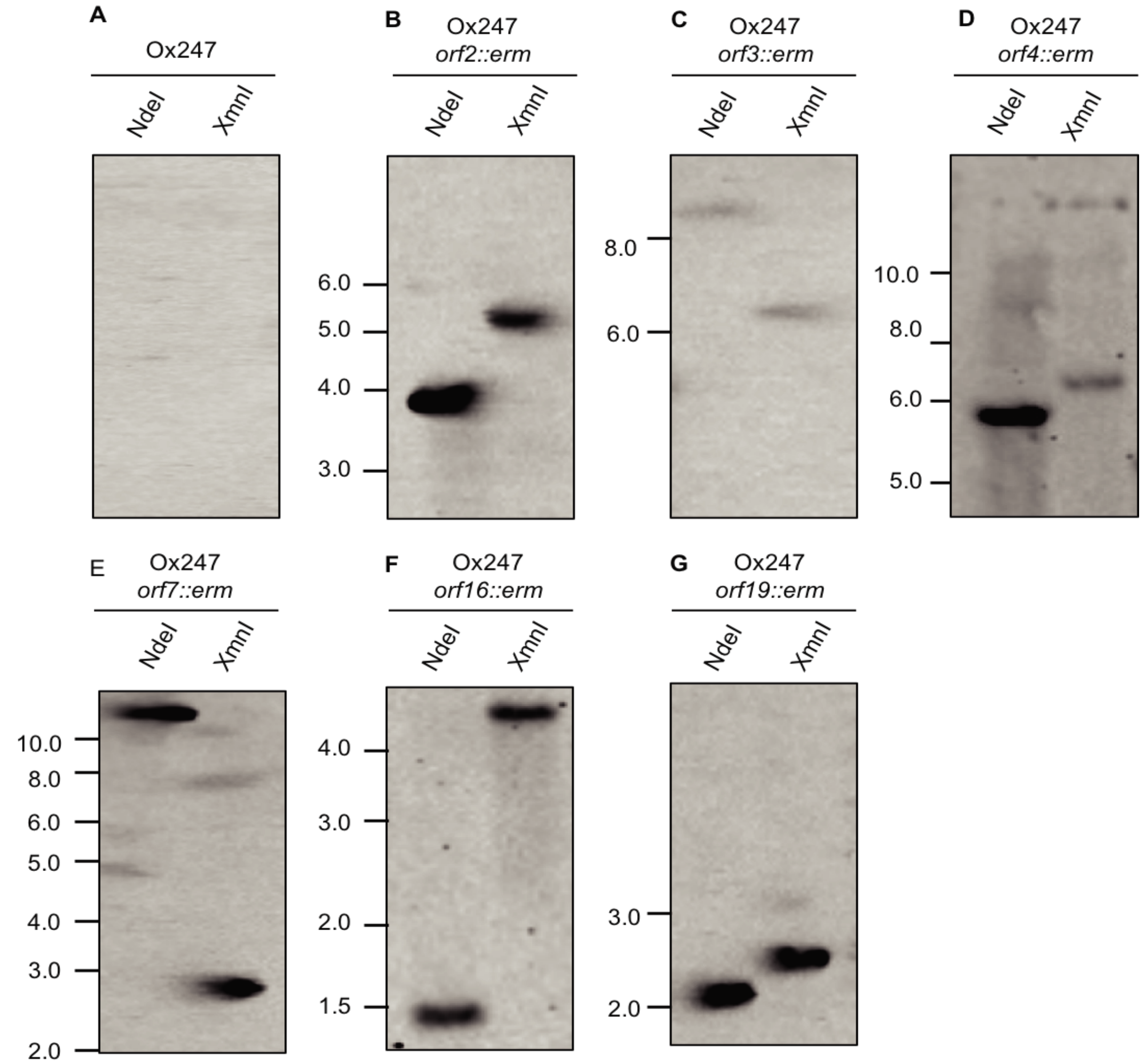

Southern blots were carried out using a probe that annealed to the 5′ end of the group II intron.
Genomic DNA was prepared and digested with NdeI and XmnI.
Expected sizes for A, Ox247, none (probe does not anneal to OX247 DNA);
B, Ox247 *orf2::erm*, ~3.9 kb, ~5.2 kb; C, Ox247 *orf3::erm*, ~6.4 kb, ~8.2kb;
D, Ox247 *orf4::erm*, ~5.9 kb, ~6.9 kb; E, Ox247 *orf7::erm*, ~2.8 kb, ~12.4 kb;
F, Ox247 *orf16::erm*,~1.3 kb, ~4.5 kb and G, Ox247 *orf19::erm*, ~2.1 kb, ~2.4 kb.

Figure S4

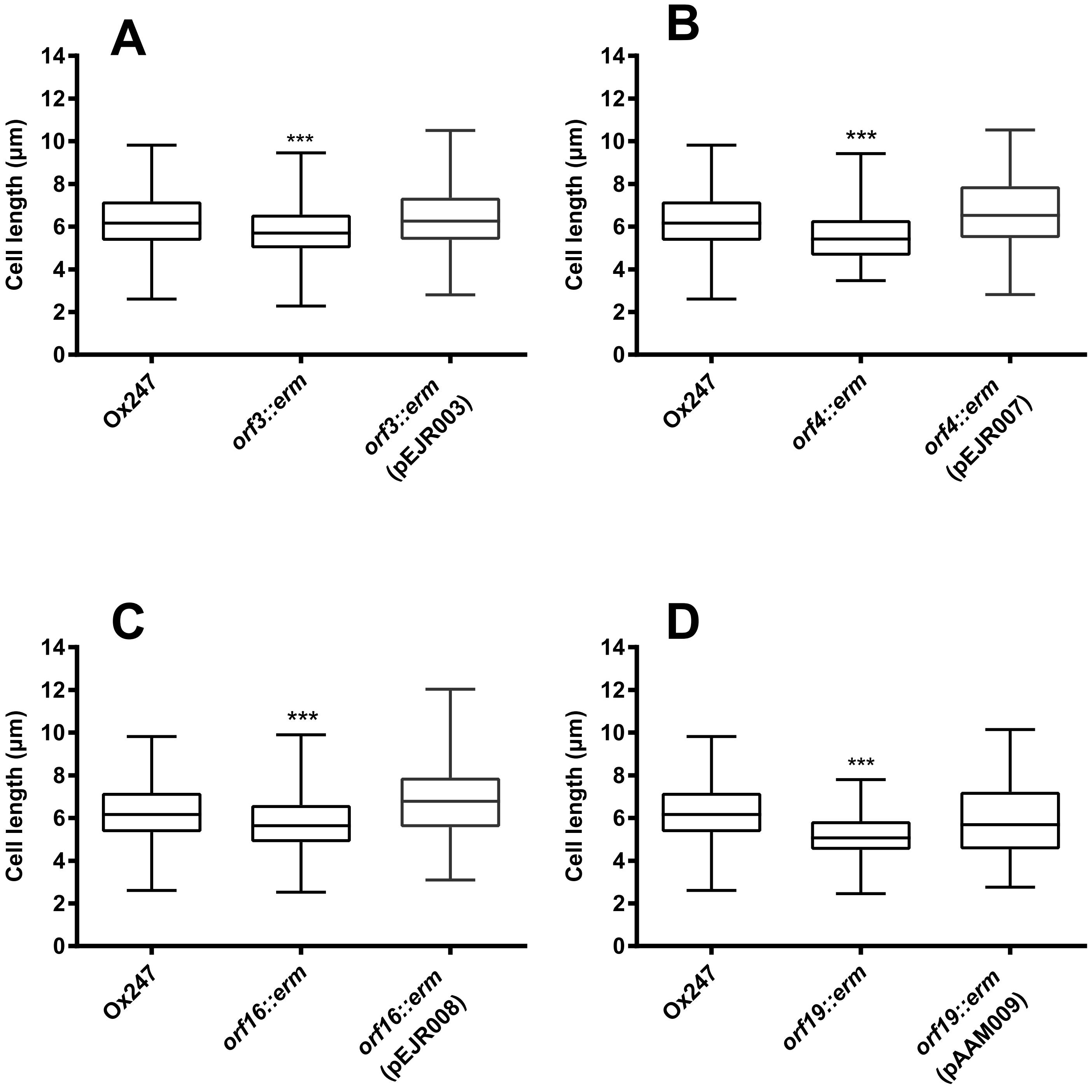

Fig S4. Complementation of cell length defect in (A), Ox247 *orf3*, (B) OX247 *orf4*, (C) Ox247 *orf16* and (D) Ox247 *orf19* mutants with the relevant plasmid expressing the wild type allele. The Ox247 data point in panel A is reused in panels B, C and D and is reused from Fig 10, because the data were acquired from the same experiment. The *orf3::erm*, *orf4::erm*, *orf16::erm* and *orf19::erm* data points are reused from Fig 10, panel A, because all the data was acquired from the same experiment.

Figure S5

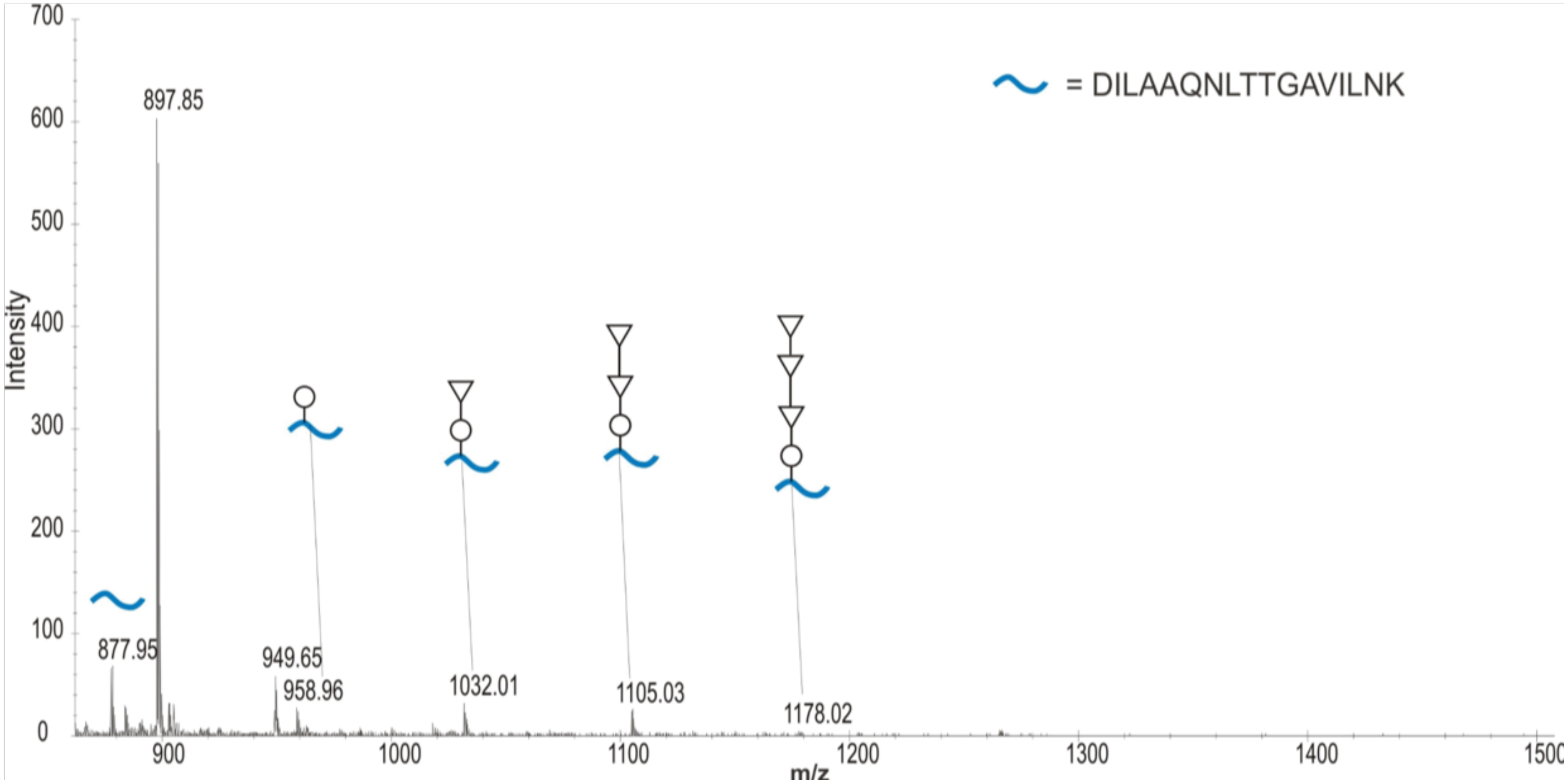

Fig S5. Summed MS data acquired at 40.2 min in the online nanoLC-ES-MS of a tryptic digest of the band at 20 kDa of Ox247 *orf4*::*erm* mutant. Expanded middle mass region to show doubly-charged peaks that corresponds to glycan compositions ranging from a single hexose up to dHex3Hex. m/z 897.95 is from elsewhere in the digest.

References

1. Dingle, K. E., Didelot, X., Ansari, M. A., Eyre, D. W., Vaughan, A., Griffiths, D., Ip, C. L., Batty, E. M., Golubchik, T., Bowden, R., Jolley, K. A., Hood, D. W., Fawley, W. N., Walker, A. S., Peto, T. E., Wilcox, M. H., and Crook, D. W. (2013) Recombinational switching of the Clostridium difficile S-layer and a novel glycosylation gene cluster revealed by large-scale whole-genome sequencing. *J Infect Dis*. **207**, 675–686
2. Novotny, R., Schaffer, C., Strauss, J., and Messner, P. (2004) S-layer glycan-specific loci on the chromosome of Geobacillus stearothermophilus NRS 2004/3a and dTDP-L-rhamnose biosynthesis potential of G. stearothermophilus strains. *Microbiology*. **150**, 953–965
3. Sebaihia, M., Wren, B. W., Mullany, P., Fairweather, N. F., Minton, N., Stabler, R., Thomson, N. R., Roberts, A. P., Cerdeno-Tarraga, A. M., Wang, H., Holden, M. T., Wright, A., Churcher, C., Quail, M. A., Baker, S., Bason, N., Brooks, K., Chillingworth, T., Cronin, A., Davis, P., Dowd, L., Fraser, A., Feltwell, T., Hance, Z., Holroyd, S., Jagels, K., Moule, S., Mungall, K., Price, C., Rabbinowitsch, E., Sharp, S., Simmonds, M., Stevens, K., Unwin, L., Whithead, S., Dupuy, B., Dougan, G., Barrell, B., and Parkhill, J. (2006) The multidrug-resistant human pathogen Clostridium difficile has a highly mobile, mosaic genome. *Nat Genet*. **38**, 779–786
4. Stabler, R. A., He, M., Dawson, L., Martin, M., Valiente, E., Corton, C., Lawley, T. D., Sebaihia, M., Quail, M. A., Rose, G., Gerding, D. N., Gibert, M., Popoff, M. R., Parkhill, J., Dougan, G., and Wren, B. W. (2009) Comparative genome and phenotypic analysis of Clostridium difficile 027 strains provides insight into the evolution of a hypervirulent bacterium. *Genome Biol*. **10**, R102
